# Obesity indices and the risk of total and cardiovascular mortality among people with diabetes: a long-term follow-up study in Taiwan

**DOI:** 10.1186/s12933-023-02072-3

**Published:** 2023-12-13

**Authors:** Chung-Yen Lu, Hsiao-Hui Chen, Kuan-Hui Chi, Pei-Chun Chen

**Affiliations:** 1 Department of Sport and Health Management, Da-Yeh University, Changhua, 515 Taiwan; 2 Long Health Chinese Medicine Clinic, Taipei, 106 Taiwan; 3 Department of Public Health, China Medical University, Taichung, 406 Taiwan; 4 Department of Medical Research, Taichung Veterans General Hospital, Taichung, 407 Taiwan; 5 National Center for Geriatrics and Welfare Research, National Health Research Institutes, 35 Keyan Road, Miaoli, 350 Taiwan; 6 Big Data Center, China Medical University Hospital, Taichung, 404 Taiwan

**Keywords:** Diabetes Mellitus, Obesity indicators, All-cause mortality, Cardiovascular mortality

## Abstract

**Background:**

The association between obesity indicators and mortality in individuals with diabetes remains unclear, and data on cardiovascular mortality are scarce. Therefore, we investigated the associations between the five adiposity indices and both all-cause and cardiovascular mortality in patients with diabetes.

**Methods:**

This cohort study included 34,686 adults with diabetes who underwent a standard health-screening program between 1996 and 2017 in Taiwan. The dates and causes of death till January 2022 were retrieved from the National Death Registry. Cox proportional hazards models were used to calculate the hazard ratios (HR) and 95% confidence intervals (CI) for all-cause and cardiovascular mortality in relation to body mass index (BMI), waist circumference, waist-hip ratio (WHR), body fat percentage (BF%), and A Body Shape Index (ABSI), using the third quintile as the reference group.

**Results:**

During a median follow-up of 15 years, there were 8,324 deaths, of which 1,748 were attributed to cardiovascular disease. After adjusting for demographics, lifestyle factors and comorbidities, ABSI was associated with all-cause mortality in an exposure-response manner; the HR (95% CI) for first and fifth vs. third quintile was 0.78 (0.69–0.89) and 1.24 (1.14–1.35), respectively. A similar but weaker exposure-response relationship was found between WHR and mortality. People with a lower BMI and BF% had an increased risk of mortality (HR [95% CI] for the first vs. third quintiles, 1.33 [1.22, 1.44] and 1.42 [1.30, 1.56], respectively). No association was observed between waist circumference categories and risk of mortality. Similar results were observed for the association of BF%, waist circumference, and ABSI with cardiovascular mortality. However, no significant association was observed between BMI and cardiovascular mortality. The association between WHR and cardiovascular mortality was stronger than that between WHR and all-cause mortality.

**Conclusions:**

ABSI demonstrated a consistent exposure-response relationship with both all-cause and cardiovascular mortality in this Asian cohort with diabetes. Our findings highlight the importance of monitoring ABSI, a surrogate index of central adiposity, in patients with diabetes.

**Supplementary Information:**

The online version contains supplementary material available at 10.1186/s12933-023-02072-3.

## Background

Analyses of diabetes trends consistently have revealed a substantial and escalating global burden of diabetes [1, 2]. In 2021, an estimated 537 million people were living with diabetes mellitus worldwide, and the age-adjusted prevalence is projected to increase to 784 million (11.2%) by 2045 in the adult population [2]. Given the substantial excess risk of death from any cause and cardiovascular diseases among individuals with diabetes, mitigating the burden of diabetes remains a public health priority [3, 4].

Obesity is a well-recognized and modifiable risk factor for the prevention and management of diabetes; however, evidence on the relationship between obesity indices and mortality in people with diabetes has been inconclusive. A meta-analysis of 24 cohorts (414,587 participants with diabetes) revealed a U-shaped association between body mass index (BMI) and all-cause mortality [5]. In contrast, the Fremantle Diabetes Study, which focused on the comparison of various obesity indices, showed that neither BMI nor waist circumference were associated with mortality in people with diabetes [6]. They found that A Body Shape Index (ABSI), a measure derived from waist circumference, body height, and BMI, was significantly associated with mortality, and the relationship was linear [6].

Although the BMI is a widely used measure of general obesity in clinical guidelines and research settings, it has limitations in reflecting body shape and regional fat deposition [7]. Waist circumference, waist-to-hip ratio (WHR), and waist-to-height ratio are well-known anthropometric proxies for central obesity, which is characterized by excess abdominal fat deposition. Growing evidence suggests that these indices of central obesity are independently associated with mortality and cardiovascular risks, and that both BMI and measures of central obesity may offer enhanced risk factor stratification compared to using either approach alone [8–10].

ABSI was designed to assess abdominal obesity without being confounded by BMI [11]. Several studies have shown that ABSIs are associated with a greater risk of all-cause mortality than BMI or waist circumference in the general population [12, 13]. However, very few studies have been conducted in people with diabetes to evaluate the association between ABSI and mortality, and data on cardiovascular mortality are lacking [6, 14]. In this study, we examined the association of five adiposity indices (BMI, waist circumference, WHR, body fat percentage, and ABSI) with the risk of death from any cause or cardiovascular disease in a large cohort of individuals with diabetes in Taiwan.

## Methods

### Study population

In this cohort study, data were obtained from the MJ research database set up by the MJ Health Management Institution, a private fee-for-service company that offers comprehensive health-screening programs through four clinics located in the northern, northwestern, central, and southern parts of Taiwan (www.mjclinic.com.tw). All four clinics used identical screening and data management procedures approved by the ISO 9001 standard. A research database has been established to study multiple aspects of chronic diseases, as well as modifiable risk factors and biomarkers,  resulting in more than 90 publications as of 2023 [15]. Details of the MJ cohort profile and data collection have been previously described [16]. In brief, this is an open and dynamic cohort that enrolled apparently healthy individuals participating in the health screening program since 1996. The information available in the research database includes sociodemographics, lifestyle variables, and medical history gathered from self-report questionnaires, as well as the results of biochemical tests of overnight-fasted blood samples, physical examination, anthropometric measurements, and functional tests.

Approximately 615,000 participants were included in this cohort from 1996 to 2017. Within the cohort, 38,933 individuals had diabetes, defined as fasting blood glucose of greater than or equal to 126 mg/dL (7.0 mmol/L), or a self-report of taking antidiabetic medications in at least one medical examination [17] (Figure S1). The date of the first examination on which the individuals met the diagnostic criteria for diabetes was set as the baseline. Participants were excluded if they had incomplete anthropometric data (n = 3,978) or were aged less than 18 years (n = 91). We also excluded participants who died within one year of follow-up to reduce the likelihood of reverse causality (n = 178). All participants provided informed consent, authorizing the MJ group for data processing. This study was approved by the Institutional Review Board of the Antai Medical Care Cooperation Antai-Tian-Sheng Memorial Hospital.

### Adiposity measurements and covariates

Details regarding the measurement of adiposity indices have been described previously [16, 18, 19]. Body weight and height were measured using an auto-anthropometer (KN-5000 A; Nakamura, Tokyo, Japan), with the participants wearing light clothing and no footwear. BMI was calculated by dividing the weight in kilograms by the square of the height in meters. Waist circumference (at the midpoint between the iliac crest and the lower end of the rib cage) and hip circumference (around the pelvis at the widest portion of the buttocks) were measured using a tape. ABSI was calculated using the following formula [11]:

ABSI = waist circumference / (BMI ^2/3^ × height^1/2^).

Body fat percentage (BF%) was measured using Bioelectric Impedance (BIA) equipment (TANITA Body Composition Analyser, TBF series, multi-frequency [5, 50, 250 and 500 kHz] model with foot-to-foot 4-electrode, TINATA, Japan). The participants were required to stand barefoot on the platform of the equipment, and the BF% was automatically calculated. The equations for estimating BF% in men and women have been described previously [19–21]. Evidence indicates variations in adipose tissue distribution between men and women, which could be attributed to inherent physiological disparities including hormonal factors, genetic determinants, muscle mass distribution, and overall physical strength [22, 23]. In men, BF% was approximated using body density, calculated as body mass divided by body volume based on Brozek’s equations [24]. Body mass can be categorized into two principal components: fat mass and fat-free mass. In women, BF% was computed based on the estimated fat-free mass, which consisted of skeletal muscle mass, body cell mass, total body water, and bone mineral mass. The BF% prediction equations provided by the manufacturer are as follows [20, 21].

In men:

Body density = 1.100696–0.107903 × Wt × Z / Ht^2^ + 0.00017 × Z.

BF%=(4.57 / body density − 4.142) × 100.

where Wt is the weight in kilograms, Ht is the height in meters, and Z is the impedance in ohms (Q).

In women:

FFM = 13.96674 + 0.348613 × Ht^2^ / Z + 0.168998 × Wt.

BF% = (Wt - FFM) / Wt × 100.

where FFM is fat-free mass in kg;

All covariates were collected at baseline. Hypertension was defined as systolic blood pressure ≥ 140 mmHg, and/or diastolic blood pressure ≥ 90 mmHg and/or receiving antihypertensive medication. Biochemical tests, including serum lipid, creatinine, and uric acid levels, were performed in accredited laboratories, as described elsewhere [16]. Chronic kidney disease was defined as an estimated glomerular filtration rate < 60 mL/min/1.73 m^2^, calculated using the Modification of Diet in Renal Disease equation as follows:186 × (serum creatinine in mg/dL) − 1.154 × (age) − 0.203 × 0.742 (if female). Leisure-time physical activity was assessed through self-reported questionnaires, with participants categorizing the intensity of their most frequent exercises over the past month based on examples provided for each intensity level. Light activity included walking, sweeping, etc.; moderate activity included brisk walking, hiking, etc.; and vigorous activity (high intensity) included activities such as running and rope skipping. Participants who reported little (less than 1 h weekly) to no exercise were categorized as sedentary.

### Outcome variables and follow-up

Death from cardiovascular disease and all causes and the date of death were determined from the National Death Registry, a dataset maintained by the Health and Welfare Data Science Center of the Ministry of Health and Welfare, Taiwan. Registration of deaths is mandatory in Taiwan. The follow-up period started at baseline and ended at death or January 12, 2022 (the currently available data). Deaths were attributed to cardiovascular disease if the underlying causes of death in the death certificates included hypertensive disease, heart disease, cerebrovascular disease, arteriolosclerosis, or aortic aneurysm and dissection (see Table S1 for the diagnosis codes).

### Statistical analysis

The participants were classified into five groups using the quintile values of adiposity measurements (Table S2). Differences in baseline characteristics among groups were examined by one-way analysis of variance (ANOVA) for continuous variables and χ^2^ test for categorical variables. Cox proportional hazards models were fitted to obtain hazard ratios (HR) and 95% confidence intervals (CI) to assess the association between the five adiposity indices and all-cause mortality with the 3rd quintile as the reference group. The unadjusted model estimated the crude association with the quintiles of adiposity indices. Model 1 was adjusted for age (years) at baseline and sex (male and female). Model 2 was additionally adjusted for other potential confounders measured at baseline, including marital status (single, married, widowed/divorced), education (illiterate, junior secondary school or below, senior secondary school, college or above), smoking (never, quit, current), alcohol consumption (never, quit, current), leisure-time physical activity (sedentary, light, moderate, and vigorous activity), and antihyperglycemic drugs. In Model 3, we added variables that might be in the causal pathway between obesity measures and mortality in Model 2, including hypertension, cardiovascular disease (including stroke), chronic kidney disease, cancer, and levels of uric acid and fasting glucose (continuous scale in mg/dL). Obesity is a well-established risk factor for cardiovascular disease [25], and individuals with cardiovascular diseases are at an increased risk of total and cardiovascular mortality. Therefore, we considered cardiovascular disease as a potential mediating variable and included it in Model 3. All the models were repeated for cardiovascular mortality. We also evaluated the exposure-response relationships of the obesity indices with the risks of all-cause and cardiovascular mortality using restricted cubic splines with four knots located at the 5th, 35th, 65th and 95th percentiles of the distribution of obesity indices.

The proportional hazard assumption was tested by including an interaction term between the obesity categories and a function of follow-up time in the regression models. This assumption was met for the association between obesity indices and cardiovascular mortality, but not for all-cause mortality. To further observe the nature of the non-proportionality over time, we conducted a regression analysis by dividing the follow-up duration into 5-year increments.

We performed two sensitivity analyses to evaluate the robustness of the results. First, the multiple imputation technique (MICE package in R) was applied under the missing-at-random assumption to address missing data on covariates and improve efficiency [26]. The Cox models were repeated using the imputed dataset. Second, the propensity score method is an alternative to the conventional covariate adjustment to mitigate selection bias and potential confounding factors. It is recommended to use alternative methods of analysis to confirm the consistency of the results, as each approach has its advantages and disadvantages [27]. We created propensity score-matched pairs by matching participants with ABSI of < 5th quintile to those with ≥ 5th ABSI quintile using the greedy-matching algorithm. Propensity scores were estimated using a logistic regression model in which all covariates in Model 2 were independent variables. Cox proportional hazards models stratified on the matched pairs were used to estimate the HRs and 95% CIs. Furthermore, we performed an exploratory stratified analysis of subgroups defined by age, sex, smoking, chronic kidney disease, cardiovascular disease, and BMI to observe whether the association between ABSI and mortality was homogeneous in the subgroups. The statistical significance of the interaction terms was tested using the likelihood ratio test.

To explore whether obesity indices improve the ability of risk prediction, we compared the performance of the base models (Models 1 and 3) versus the models that added each obesity indicator to the base model for predicting mortality. Three performance measures were computed: c-statistic, integrated discrimination improvement (IDI), and the continuous net reclassification improvement (NRI) [28].

We used SAS version 9.4 (SAS Institute Inc., Cary, NC, USA) and R version 4.2.3 (R Foundation for Statistical Computing, Vienna, Austria) for statistical analyses. The significance level for the statistical tests was set at p < 0.05.

## Results

### Baseline characteristics of participants

Of the 38,933 individuals with diabetes, 34,686 met the inclusion criteria and were included in the study (Figure S1). Descriptive statistics of the five adiposity indices, including the cutoff values of the quintiles, are presented in Table S2. Table 1 shows the baseline characteristics of the participants according to the quintile categories of the ABSI. Participants in the higher ABSI quintile group were more likely to be men, older, ever-smokers, ever-drinkers, have a higher prevalence of chronic diseases, and have higher fasting glucose levels. In addition, they tended to have lower levels of education.

**Table 1:** Baseline characteristics of subjects with diabetes according to ABSI category

Variables	ABSI, m^11/6^ kg^− 2/3^ (n = 34,686)					P value
	< 0.073	0.073 to < 0.076	0.076 to < 0.078	0.078 to < 0.081	≥ 0.081	
	(n = 5,959)	(n = 7,463)	(n = 6,105)	(n = 7,629)	(n = 7,530)	
Sex, n (%)						< 0.0001
Men	1,494(25.07)	3,537(47.39)	3,812(62.44)	5,318(69.71)	5,354(71.10)	
Women	4,465(74.93)	3,926(52.61)	2,293(37.56)	2,311(30.29)	2,176(28.90)	
Age, mean (SD), years	47.42(12.31)	50.82(11.85)	52.81(11.64)	55.69(11.34)	61.00(10.97)	< 0.0001
18–44	2,413(40.49)	2,325(31.15)	1,572(25.75)	1,352(17.72)	600(7.97)	
45–64	3,107(52.14)	4,227(56.64)	3,587(58.76)	4,564(59.82)	3,970(52.72)	
≥ 65	439(7.37)	911(12.21)	946(15.50)	1,713(22.45)	2,960(39.31)	
Marital status, n (%)						< 0.0001
Single	832(13.96)	597(8.00)	397(6.50)	316(4.14)	161(2.14)	
Married	3,971(66.64)	5,358(71.79)	4,535(74.28)	5,824(76.34)	5,669(75.29)	
Widowed/Divorce	709(11.90)	922(12.35)	700(11.47)	909(11.92)	1,166(15.48)	
Unknown	447(7.50)	586(7.85)	473(7.75)	580(7.60)	534(7.09)	
Education, n (%)						< 0.0001
Illiterate	378(6.34)	599(8.03)	488(7.99)	638(8.36)	1,024(13.60)	
Junior secondary school or below	1,847(31.00)	2,417(32.39)	1,995(32.68)	2,723(35.69)	3,225(42.83)	
Senior secondary school	1,255(21.06)	1,502(20.13)	1,171(19.18)	1,479(19.39)	1,245(16.53)	
College or above	2,176(36.52)	2,572(34.46)	2,147(35.17)	2,393(31.37)	1,621(21.53)	
Unknown	303(5.08)	373(5.00)	304(4.98)	396(5.19)	415(5.51)	
Smoking, n (%)						< 0.0001
Never	4,774(80.11)	5,142(68.90)	3,735(61.18)	4,280(56.10)	3,963(52.63)	
Quit	237(3.98)	499(6.69)	563(9.22)	855(11.21)	981(13.03)	
Current	646(10.84)	1,379(18.48)	1,464(23.98)	2,055(26.94)	2,109(28.01)	
Unknown	302(5.07)	443(5.94)	343(5.62)	439(5.75)	477(6.33)	
Alcohol drinking, n (%)						< 0.0001
Never	4,678(78.50)	5,406(72.44)	4,089(66.98)	4,792(62.81)	4,483(59.54)	
Quit	175(2.94)	276(3.70)	296(4.85)	460(6.03)	592(7.86)	
Current	531(8.91)	1,059(14.19)	1,185(19.41)	1,664(21.81)	1,752(23.27)	
Unknown	575(9.65)	722(9.67)	535(8.76)	713(9.35)	703(9.34)	
Leisure time physical activity, n (%)						< 0.0001
Sedentary	132(2.22)	156(2.09)	133(2.18)	151(1.98)	110(1.46)	
Light activity	3,724(62.49)	4,551(60.98)	3,597(58.92)	4,494(58.91)	4,501(59.77)	
Moderate activity	728(12.22)	900(12.06)	751(12.30)	879(11.52)	576(7.65)	
Vigorous activity	397(6.66)	491(6.58)	449(7.35)	529(6.93)	408(5.42)	
Unknown	978(16.41)	1,365(18.29)	1,175(19.25)	1,576(20.66)	1,935(25.70)	
Chronic kidney disease, n (%)	401(6.73)	646(8.66)	634(10.38)	967(12.68)	1,496(19.87)	< 0.0001
Hypertension, n (%)	1,766(29.64)	2,477(33.19)	2,113(34.61)	2,854(37.41)	3,205(42.56)	< 0.0001
Anti-hyperglycemic drugs, n (%)	2,413(40.49)	2,836(38.00)	2,345(38.41)	3,118(40.87)	3,501(46.49)	< 0.0001
Cancer, n (%)	159(2.67)	189(2.53)	152(2.49)	200(2.62)	240(3.19)	0.070
Cardiovascular disease, n (%)	399(6.70)	528(7.07)	503(8.24)	762(9.99)	1,017(13.51)	< 0.0001
Stroke, n (%)	37(0.62)	59(0.79)	72(1.18)	128(1.68)	227(3.01)	< 0.0001
Uric acid, mg/dL	5.81(1.60)	6.10(1.63)	6.21(1.66)	6.27(1.68)	6.30(1.73)	< 0.0001
Triglycerides, mg/dL^*^	4.87(0.63)	5.03(0.62)	5.09(0.63)	5.11(0.63)	5.14(0.60)	< 0.0001
Total cholesterol, mg/dL	204.58(41.29)	209.80(42.77)	211.02(43.45)	209.78(43.43)	211.27(45.64)	< 0.0001
Fasting glucose, mg/dL	148.76(53.72)	157.36(55.40)	161.35(56.58)	164.38(57.92)	168.07(61.50)	< 0.0001

Abbreviation: ABSI, A Body Shape Index

^*^Triglyceride values were log-transformed.

### Association between adiposity indices and all-cause mortality

During a median follow-up period of 14.8 years, 8,324 deaths from any cause and 1,748 deaths from cardiovascular disease were reported. All-cause mortality rose from 6.67 per 1,000 person-years in the first ABSI quintile to 31.67 per 1,000 person-years in the fifth quintile (Table 2). Similarly, all-cause mortality showed an upward trend with increasing quintiles for waist circumference and WHR. However, individuals in the lower BMI and BF% quintiles exhibited significantly higher mortality rates than those in the higher quintiles.

After adjusting for age, sex, and other potential confounders in Model 2, ABSI was associated with mortality in an exposure-response manner (Table 2). The HR (95% CI) was 0.74 (0.65, 0.84) for the first ABSI quintile and 1.28 (1.17, 1.39) for the fifth quintile compared with the third quintile. Similarly, there was a positive association between WHR and mortality (HR [95%CI], first and fifth vs. third quintile, 0.84 [0.76, 0.94] and 1.15 [1.05, 1.26], respectively), although the association was weaker than that observed for ABSI. In contrast, participants in the lower BF% quintile exhibited an increased risk of mortality. The association between BMI and mortality appeared to follow a U-shaped pattern; the HR (95% CI) was 1.29 (1.18, 1.40) for the first quintile and 1.16 (1.06, 1.27) for the fifth quintile, as compared to the third quintile. In Model 3, additionally adjusting for chronic diseases, such as hypertension, cardiovascular disease, and kidney disease, did not materially change the HRs (95% CIs) for the ABSI and BF% categories (Model 3, Table 2). The HR of mortality in the first quintile vs. the third quintile of both BMI and WHR also remained similar in Model 3, but the elevated risks associated with the fifth quintile of BMI and WHR were weakened to a borderline significance level. The association between waist circumference and the risk of all-cause mortality substantially diminished in model 3.

Spline regression analysis revealed a U-shaped association between BMI and all-cause mortality (Figure S2). Mortality risk increased progressively with increasing ABSI levels, exhibiting a steeper slope at high ABSI values. High waist circumference and WHR were positively associated with mortality. In contrast, there was an inverse association with mortality when BF% was < 35%.

**Table 2:** Hazard ratios of death from all causes in association with adiposity variables among people with diabetes

	No. of deaths	Follow-up person-years	Mortality^*^	Hazard ratio (95% confidence interval)			
				Unadjusted model	Model 1	Model 2	Model 3
BMI, kg/m^2^							
1st quintile	2,029	102,699	19.76	1.27 (1.19, 1.35)	1.35 (1.26, 1.44)	1.29 (1.18, 1.40)	1.33 (1.22, 1.44)
2nd quintile	1,855	102,858	18.03	1.15 (1.07, 1.23)	1.10 (1.02, 1.17)	1.08 (0.99, 1.17)	1.07 (0.98, 1.16)
3rd quintile	1,636	103,829	15.76	1.00 (Ref.)	1.00 (Ref.)	1.00 (Ref.)	1.00 (Ref.)
4th quintile	1,531	100,874	15.18	0.98 (0.92, 1.05)	1.10 (1.02, 1.18)	1.09 (0.99, 1.19)	1.06 (0.97, 1.16)
5th quintile	1,273	97,769	13.02	0.86 (0.80, 0.93)	1.18 (1.10, 1.27)	1.16 (1.06, 1.27)	1.11 (1.01, 1.22)
Waist circumference, cm							
1st quintile	1,210	91,959	13.16	0.77 (0.71, 0.83)	1.03 (0.95, 1.11)	0.99 (0.90, 1.09)	1.06 (0.96, 1.17)
2nd quintile	1,604	102,613	15.63	0.91 (0.85, 0.97)	0.94 (0.87, 1.00)	0.93 (0.86, 1.01)	0.95 (0.88, 1.04)
3rd quintile	1,739	101,872	17.07	1.00 (Ref.)	1.00 (Ref.)	1.00 (Ref.)	1.00 (Ref.)
4th quintile	1,775	104,516	16.98	1.01 (0.94, 1.07)	0.96 (0.90, 1.03)	0.96 (0.89, 1.05)	0.97 (0.89, 1.05)
5th quintile	1,996	107,069	18.64	1.13 (1.06, 1.21)	1.16 (1.09, 1.24)	1.11 (1.02, 1.20)	1.07 (0.99, 1.17)
Waist-hip ratio							
1st quintile	975	103,956	9.38	0.58 (0.53, 0.63)	0.79 (0.72, 0.86)	0.84 (0.76, 0.94)	0.90 (0.81, 1.01)
2nd quintile	1,662	112,865	14.73	0.90 (0.84, 0.97)	0.94 (0.88, 1.01)	0.99 (0.90, 1.08)	0.99 (0.90, 1.08)
3rd quintile	1,293	81,217	15.92	1.00 (Ref.)	1.00 (Ref.)	1.00 (Ref.)	1.00 (Ref.)
4th quintile	2,297	123,804	18.55	1.18 (1.10, 1.26)	1.12 (1.05, 1.20)	1.13 (1.03, 1.23)	1.09 (1.00, 1.19)
5th quintile	2,097	86,186	24.33	1.56 (1.46, 1.67)	1.24 (1.15, 1.33)	1.15 (1.05, 1.26)	1.07 (0.97, 1.17)
Body fat, %							
1st quintile	2,507	98,578	25.43	1.86 (1.74, 1.99)	1.42 (1.32, 1.52)	1.34 (1.23, 1.46)	1.42 (1.30, 1.56)
2nd quintile	1,731	106,137	16.31	1.20 (1.11, 1.28)	1.13 (1.05, 1.21)	1.12 (1.03, 1.23)	1.19 (1.09, 1.30)
3rd quintile	1,386	100,924	13.73	1.00 (Ref.)	1.00 (Ref.)	1.00 (Ref.)	1.00 (Ref.)
4th quintile	1,410	101,107	13.95	1.02 (0.95, 1.10)	0.99 (0.91, 1.06)	0.97 (0.88, 1.07)	0.98 (0.89, 1.08)
5th quintile	1,290	101,282	12.74	0.94 (0.87, 1.02)	0.94 (0.87, 1.02)	0.93 (0.84, 1.03)	0.91 (0.82, 1.01)
ABSI, m^11/6^ kg^− 2/3^							
1st quintile	601	90,046	6.67	0.50 (0.45, 0.55)	0.73 (0.66, 0.81)	0.74 (0.65, 0.84)	0.78 (0.69, 0.89)
2nd quintile	1,157	110,893	10.43	0.78 (0.72, 0.85)	0.89 (0.82, 0.96)	0.90 (0.82, 1.00)	0.92 (0.83, 1.02)
3rd quintile	1,182	89,257	13.24	1.00 (Ref.)	1.00 (Ref.)	1.00 (Ref.)	1.00 (Ref.)
4th quintile	2,002	111,029	18.03	1.35 (1.25, 1.45)	1.11 (1.03, 1.20)	1.08 (0.99, 1.18)	1.05 (0.96, 1.15)
5th quintile	3,382	106,804	31.67	2.35 (2.20, 2.51)	1.42 (1.33, 1.52)	1.28 (1.17, 1.39)	1.24 (1.14, 1.35)

Abbreviations: ABSI, A Body Shape Index; BMI, body mass index.

^*^Per 1000 person-years

Model 1 was adjusted for age and sex; model 2 was adjusted for age, sex, marital status, education, smoking, alcohol drinking, leisure time physical activity, and anti-hyperglycemic drugs (n = 24,403); Model 3 included Model 2 variables plus fasting glucose, hypertension, uric acid, cardiovascular disease (including stroke), chronic kidney disease, and cancer (n = 24,257).

### Association between adiposity indices and cardiovascular mortality

Table 3 shows the HRs of cardiovascular mortality associated with the categories of adiposity measures, using the third quintile as the reference. During the follow-up period, 1748 deaths were attributed to cardiovascular diseases, including 183 (10.47%) hypertensive disease, 989 (56.58%) heart disease, 817 (46.74%) cerebrovascular disease, 3 (0.17%) arteriolosclerosis, and 17 (0.97%) aortic dissection or aneurysm. Cardiovascular mortality increased gradually from the first to fifth quintiles of WHR and ABSI. Participants with a lower BMI and BF% had higher cardiovascular mortality rates. In the model adjusted for age, sex, and other potential confounders, no significant association was observed between the BMI and cardiovascular mortality (Model 2; Table 3). Individuals in the fifth quintile of waist circumference or those in the second quintile of BF% had an elevated risk of cardiovascular mortality compared to those in the third quintile (adjusted HR [95% CI], 1.24 [1.05, 1.48], and 1.25 [1.03, 1.52], respectively). HRs increased with increasing quintiles of WHR and ABSI. In Model 3, similar patterns of BMI, BF%, and ABSI were observed. However, the association between waist circumference and cardiovascular mortality was significantly weakened. Regarding WHR, the HRs in the fifth quintile decreased substantially to a statistically non-significant level. The exposure-response curves revealed results similar to those observed in the quintile analyses, whereas the CIs were wide at extreme levels of obesity indices (Figure S3).

We conducted a supplementary analysis to observe the relationship between the obesity indices and deaths related to heart and cerebrovascular diseases, the two major causes of cardiovascular mortality (Table S3). The findings were generally similar to those of overall cardiovascular mortality. Notably, the association between ABSI and cerebrovascular disease-related mortality was stronger than that with heart disease-related mortality. Furthermore, a significantly reduced risk of cerebrovascular disease mortality was found in the fifth quintile of BMI and BF% compared with the first quintile, but no such association was observed for heart disease mortality.

### Analysis stratified by follow-up duration

Figures S4–S8 show the HRs for mortality across the quintiles of BMI, waist circumference, WHR, BF%, and ABSI stratified by follow-up duration in the models adjusted for all covariates. The elevated risk of all-cause mortality associated with a low BMI and BF% persisted throughout the 15-year follow-up period (Figures S4 and S6). ABSI was the sole obesity measure that consistently exhibited an exposure-response relationship with both all-cause and cardiovascular mortality, persisting for 15 and 10 years, respectively (Figure S8).

**Table 3:** Hazard ratios of cardiovascular mortality in association with adiposity variables among people with diabetes

	No. of deaths	Follow-up person-years	Mortality^*^	Hazard ratio (95% confidence interval)			
				Unadjusted model	Model 1	Model 2	Model 3
BMI, kg/m^2^							
1st quintile	400	102,699	3.89	1.13 (0.98, 1.31)	1.21 (1.05, 1.39)	1.06 (0.89, 1.27)	1.18 (0.98, 1.41)
2nd quintile	380	102,858	3.69	1.07 (0.92, 1.23)	1.02 (0.88, 1.18)	0.92 (0.77, 1.09)	0.92 (0.77, 1.10)
3rd quintile	360	103,829	3.47	1.00 (Ref.)	1.00 (Ref.)	1.00 (Ref.)	1.00 (Ref.)
4th quintile	319	100,874	3.16	0.93 (0.80, 1.08)	1.05 (0.90, 1.22)	1.06 (0.88, 1.28)	1.01 (0.84, 1.22)
5th quintile	289	97,769	2.96	0.89 (0.76, 1.04)	1.25 (1.07, 1.45)	1.16 (0.96, 1.42)	1.06 (0.87, 1.29)
Waist circumference, cm							
1st quintile	222	91,959	2.41	0.69 (0.58, 0.81)	0.93 (0.78, 1.10)	0.79 (0.64, 0.98)	0.90 (0.72, 1.12)
2nd quintile	339	102,613	3.30	0.93 (0.80, 1.08)	0.96 (0.83, 1.12)	0.86 (0.72, 1.04)	0.90 (0.75, 1.08)
3rd quintile	357	101,872	3.50	1.00 (Ref.)	1.00 (Ref.)	1.00 (Ref.)	1.00 (Ref.)
4th quintile	365	104,516	3.49	1.01 (0.87, 1.17)	0.96 (0.83, 1.11)	0.94 (0.78, 1.12)	0.91 (0.76, 1.09)
5th quintile	465	107,069	4.34	1.29 (1.12, 1.48)	1.32 (1.15, 1.51)	1.24 (1.05, 1.48)	1.13 (0.95, 1.34)
Waist-hip ratio							
1st quintile	162	103,956	1.56	0.45 (0.37, 0.55)	0.61 (0.50, 0.75)	0.60 (0.47, 0.78)	0.67 (0.51, 0.86)
2nd quintile	334	112,865	2.96	0.86 (0.73, 1.01)	0.89 (0.76, 1.05)	0.92 (0.75, 1.13)	0.92 (0.74, 1.12)
3rd quintile	273	81,217	3.36	1.00 (Ref.)	1.00 (Ref.)	1.00 (Ref.)	1.00 (Ref.)
4th quintile	522	123,804	4.22	1.27 (1.10, 1.47)	1.21 (1.05, 1.41)	1.31 (1.09, 1.58)	1.23 (1.02, 1.48)
5th quintile	457	86,186	5.30	1.61 (1.39, 1.88)	1.28 (1.10, 1.50)	1.34 (1.10, 1.63)	1.15 (0.94, 1.40)
Body fat, %							
1st quintile	503	98,578	5.10	1.82 (1.57, 2.10)	1.31 (1.12, 1.53)	1.21 (0.99, 1.47)	1.35 (1.11, 1.65)
2nd quintile	391	106,137	3.68	1.31 (1.13, 1.53)	1.20 (1.03, 1.41)	1.25 (1.03, 1.52)	1.38 (1.14, 1.67)
3rd quintile	285	100,924	2.82	1.00 (Ref.)	1.00 (Ref.)	1.00 (Ref.)	1.00 (Ref.)
4th quintile	304	101,108	3.01	1.07 (0.91, 1.26)	1.06 (0.90, 1.26)	1.07 (0.87, 1.32)	1.06 (0.86, 1.31)
5th quintile	265	101,282	2.62	0.94 (0.80, 1.11)	0.99 (0.82, 1.18)	0.95 (0.76, 1.20)	0.88 (0.70, 1.10)
ABSI, m^11/6^ kg^− 2/3^							
1st quintile	121	90,046	1.34	0.47 (0.38, 0.58)	0.70 (0.56, 0.87)	0.71 (0.54, 0.93)	0.74 (0.56, 0.97)
2nd quintile	215	110,893	1.94	0.68 (0.56, 0.81)	0.78 (0.65, 0.93)	0.70 (0.55, 0.88)	0.69 (0.55, 0.88)
3rd quintile	253	89,257	2.83	1.00 (Ref.)	1.00 (Ref.)	1.00 (Ref.)	1.00 (Ref.)
4th quintile	420	111,029	3.78	1.32 (1.13, 1.54)	1.08 (0.92, 1.26)	1.06 (0.87, 1.29)	1.02 (0.84, 1.24)
5th quintile	739	106,804	6.92	2.40 (2.08, 2.76)	1.41 (1.22, 1.64)	1.31 (1.09, 1.57)	1.23 (1.02, 1.48)

Abbreviations: ABSI, A Body Shape Index; BMI, body mass index

^*^Per 1000 person-years

Model 1 was adjusted for age and sex; model 2 was adjusted for age, sex, marital status, education, smoking, alcohol drinking, leisure time physical activity, and anti-hyperglycemic drugs (n = 24,403); Model 3 included Model 2 variables plus fasting glucose, hypertension, uric acid, cardiovascular disease (including stroke), chronic kidney disease, and cancer (n = 24,257).

### Sensitivity and subgroup analysis

In our analysis, the percentages of missing covariate values generally ranged from 5 to 9%, except for physical activity, for which data were missing for 20% of the participants (Table 1). The percentages of missing values among the ABSI quintiles for baseline characteristics were similar, except for leisure-time physical activity, where the percentage of missing values was higher in individuals with a higher ABSI (Table S4). Further analysis showed that 69.9% of all participants had complete data for all variables, and 21.5% had missing values for only one variable. To improve the efficiency of the multiple regression analysis (model 3 in Tables 2 and 3), we performed a sensitivity analysis using multiple imputation techniques and found similar patterns of results for the association between all adiposity indices and all-cause mortality (Table S5). The results for cardiovascular mortality also did not change materially, except that a significantly increased risk was observed in the first BMI quintile and the elevated risk associated with high WHR decreased substantially (Table S6).

In the sensitivity analysis where propensity score matching was applied, there were 4551 matched pairs of people with an ABSI < 5th and ≥ 5th quintile (Table S7). Distributions of all matched variables were similar between the two groups. The association between ABSI and all-cause and cardiovascular mortality in the original sample was consistent with that in the matched sample, although the HRs were slightly lower in the matched sample (Table S8).

Analyses stratified by age, sex, smoking status, chronic kidney disease, and cardiovascular disease consistently revealed an increased risk of all-cause mortality associated with elevated ABSI values in all subgroups (Table S9). An exception was that in people aged 18–44 years, the HRs fluctuated across the quintiles. In all subgroups, the HRs were lowest in the first ABSI quintile and highest in the fifth ABSI quintile compared to the third quintile. The association between the ABSI and all-cause mortality was stronger in women than in men (P for interaction = 0.010). No statistical interaction was observed between the ABSI and age, smoking status, chronic kidney disease, or cardiovascular disease. In the analysis stratified by BMI, participants in the fifth quintile generally had an increased risk of all-cause mortality compared to those in the third quintile. ABSI was associated with all-cause mortality in an exposure-response manner in all BMI subgroups, except for the fourth BMI quintile, where the HRs that fluctuated across the quintile groups were not statistically significant (P for interaction = 0.001).

In the stratified analysis, a positive association was observed between ABSI and cardiovascular mortality (Table S10). The highest HRs were observed in the fifth ABSI quintile compared with the third ABSI quintile in all subgroups, except for those with pre-existing cardiovascular disease. Notably, the positive association between ABSI and cardiovascular mortality appeared to be stronger in the fifth BMI quintile than in the other quintiles. However, the likelihood ratio test did not reveal any significant interactions in any stratified analysis.

To observe whether the association between ABSI and mortality varied among people with and without weight changes, we performed an additional analysis restricted to participants with at least two measurements of body weight (n = 14,223). Weight changes were categorized into two groups: ≥5% and < 5%. In both groups, we found a positive association between the ABSI and the risk of both total and cardiovascular mortality (Table S11). The associations did not differ between the groups with weight changes of ≥ 5% and < 5% (P for interaction, 0.94 for all-cause mortality, and 0.99 for cardiovascular mortality, respectively). In both groups, HRs were highest in the fifth ABSI quintile when compared with the third quintile and were statistically significant.

### Model performance in predicting mortality

The c-statistic of Model 3 was 0.785 for all-cause mortality (Table S12). The C-statistics increased slightly when BMI (0.787), BF% (0.788), or ABSI (0.787) were added to Model 3, but the increase was not significant. Among the obesity indices, ABSI offered the greatest improvement in the prediction of all-cause mortality when considering both continuous NRI (0.173) and IDI (0.0016), followed by WHR (continuous NRI, 0.174; IDI, 0.0004). In the analysis where Model 1 served as the base model in place of Model 3 (Table S12), the C-statistic was 0.747. Similar to the findings for Model 3, the obesity indices had little effect on the C-statistic, and ABSI resulted in the greatest increase in both the continuous NRI (0.257) and IDI (0.0044).

For the prediction of cardiovascular mortality, Model 3 yielded a C-statistic of 0.811 (Table S12). The C-statistics increased the most with the addition of WHR (0.814) or ABSI (0.814); however, the increase was not significant. Adding ABSI to Model 3 led to the greatest IDI (0.0012), whereas WHR and BF% offered higher NRI than ABSI (continuous NRI: 0.245, 0.220, and 0.184, respectively). Similar findings were obtained when Model 1 was used as the base model.

## Discussion

In the present study of over 38,000 people with diabetes, we investigated the associations of five commonly used measures of adiposity and long-term mortality from any cause and cardiovascular disease. After considering several confounders, there was a consistent exposure-response relationship between ABSI and all-cause and cardiovascular mortality. These associations persisted for 10 to 15 years of follow-up. Conversely, an increased risk of all-cause and cardiovascular mortality was observed in individuals in the lowest quintiles of BMI and BF% compared to those in the 3rd quintile.

In line with our observations, previous studies involving primarily white ethnic populations have consistently reported a positive relationship between ABSI and all-cause mortality in people with diabetes [6, 14, 29]. In the Fremantle Diabetes Study of Australian patients with type 2 diabetes, people in the fifth ABSI quintile had an elevated risk of all-cause mortality compared with those in the first quintile [6]. ABSI is the obesity indicator most strongly associated with all-cause mortality when compared with BMI, waist circumference, and WHR in their analysis [6]. Similarly, in a prospective cohort study on Italian Caucasians, the highest risk was found in the third ABSI tertile group [14]. However, none of these studies have reported data on cardiovascular mortality. Among adults with diabetes in the United States, an elevated ABSI was linearly associated with the risk of all-cause mortality [29]. In their analysis, stratified by ethnicity, a significant association between ABSI and cardiovascular mortality was found only in Mexican Americans, suggesting a potential ethnic difference [29]. However, the data for the ethnic Chinese and Asian subgroups were not provided separately. Potential ethnic differences in these associations warrant further investigation.

The ABSI provides conceptual advantages and physiological aspects for its association with mortality. A high ABSI reflects a more centralized concentration of body shape measured by waist circumference for a given body size measured by weight and height [11]. There is growing recognition that waist circumference allows for a more refined risk stratification for future morbidity and mortality, complementing BMI [29]. Furthermore, previous studies reported that the positive association between waist circumferences and mortality became fully evident only after adjusting for BMI [30, 31]. Using ABSI allows us to establish the independent contribution of waist circumference, and avoids potential collinearity issues between BMI and waist circumference in the regression adjustments [11, 32]. From a physiological perspective, a high ABSI may reflect excess visceral (abdominal) fat compared to the peripheral tissue. In patients with diabetes, ABSI has been positively correlated with visceral fat [33], which was identified as an independent risk factor for all-cause mortality and incidence of cardiovascular disease [34, 35].

The ‘obesity-survival paradox’ in people with chronic diseases has been debated over the past decade [30]. This phenomenon refers to the counterintuitive observation that despite the known association between obesity and increased mortality, overweight and obese individuals tend to have better survival than leaner individuals within populations with chronic diseases, such as heart disease, hypertension, and diabetes [31, 32]. A growing body of evidence suggested that the ‘obesity-survival paradox’ may be attributed to several methodological issues [30, 32]. One of the primary sources of bias is misclassification resulting from using BMI as an index of obesity [14, 32]. The BMI does not distinguish between central (visceral) and peripheral (subcutaneous) fat distributions, which have differential effects on health. Visceral fat is causally related to survival, whereas subcutaneous fat seems less relevant in determining disease risk [33]. After controlling for several potential confounders, our analysis revealed evidence of an obesity paradox with BMI; however, no such phenomenon was observed with ABSI. These data highlight the prognostic importance of the ABSI, derived from a surrogate measure of visceral adiposity, in diabetes.

Previous studies generally indicated an independent association between body fat and all-cause mortality in a J or U shaped manner in the general population [19, 34]. However, we are unaware of any cohort study reporting this issue in patients with diabetes. Our analysis showed that low BF% was associated with an increased risk of both all-cause and cardiovascular mortality (first and second vs. third quintile). In the present study, BF% was estimated using BIA, a noninvasive and simple method that relies on the assumption of constant hydration of fat-free mass [35]. However, this assumption may not be valid in patients with diabetes, as treatment for glucose control could result in alterations in water retention and distribution as well as in bone and fat mass [35–37]. Compared to Dual-energy X-ray absorptiometry, an advanced method for monitoring body composition, BIA may overestimate both fat mass and fat mass % in patients with diabetes [38]. Furthermore, evidence has shown that in patients with diabetes and obesity, hydration of fat-free mass decreases with weight loss [35]. Hence, the utility of BIA in assessing body composition in patients with diabetes needs to be verified.

The reduction in the association between obesity measures and the risk of mortality over time has been observed in various patient populations [39, 40]. In a study involving patients on dialysis, both the reduced risk of mortality associated with low BMI and the increased risk of mortality associated with high BMI disappeared approximately seven years after initiating dialysis [39]. The association between obesity and mortality may be influenced by other factors, particularly over a long-term follow-up period. For example, overweight and obese individuals may have made lifestyle changes and focused more on managing their comorbidities. Additionally, in people with diabetes, competitive risk factors such as diabetes-related complications might have a more pronounced impact on survival than obesity.

In the supplementary analysis evaluating model performance in predicting mortality, we observed that the addition of ABSI or other obesity indices did not enhance the discrimination ability (i.e., c-statistic). ABSI may provide a modest improvement in risk stratification for predicting mortality. However, it is noteworthy that our comparison is a supplementary analysis. Further studies specifically designed to develop and compare prediction models using ABSI as a predictor in patients with diabetes are needed to confirm our observations.

The present study included a large cohort of people with diabetes, and the comprehensive assessment of potential confounders, such as smoking, physical activity, and comorbidities, helped mitigate the issues of collider bias and reverse causation [14, 32]. Long-term follow-up allowed us to observe whether these associations changed over time. This study has several limitations that need to be acknowledged. First, we were unable to accurately identify patients with type 1 or type 2 diabetes mellitus. However, the findings of the present study should apply to people with type 2 diabetes because, among adults in Taiwan, people with type 1 diabetes account for only 0.5–0.6% of the population with diabetes [41]. Second, unmeasured confounding might have occurred because data on some potential confounders such as diet, duration of diabetes, use of insulin, type of antidiabetic drugs, and medication adherence were not available. Third, unintentional weight loss may result in a reverse causation. To reduce the impact, the regression models were adjusted for several comorbidities, including cancer, cardiovascular disease, stroke, and chronic kidney disease. Furthermore, in the analysis stratified by follow-up duration, the associations persisted after 5 years of follow-up. Fourth, the study participants may have had a better socioeconomic status than the general population because the MJ cohort consisted of people who received self-paid or occupational health examinations. Therefore, our findings may not be representative of the entire Taiwanese population in terms of socioeconomic status.

In conclusion, this study suggests that in an Asian cohort with diabetes, there is evidence of an obesity paradox with BMI and BF%, but this phenomenon was not observed for ABSI, a surrogate index of central adiposity. There was an exposure-response association between ABSI and the risk of both all-cause and cardiovascular mortality, which was consistently observed over a 10-year follow-up period. Our findings emphasize the importance of ABSI monitoring in the care of patients with diabetes.

### Electronic supplementary material

Below is the link to the electronic supplementary material.

Supplementary Material 1

## Data Availability

The datasets used and/or analyzed in the current study are available from the corresponding author upon reasonable request.

## References

[1] Global regional, national burden of diabetes. and From 1990 to 2021, with projections of prevalence to 2050: a systematic analysis for the global burden of Disease Study 2021. Lancet. 2023. 10.1016/S0140-6736(23)01301-6 PMC10364581 37356446

[2] International Diabetes Federation, Facts. & figures [https://idf.org/about-diabetes/facts-figures/].

[3] An Y, Zhang P, Wang J, Gong Q, Gregg EW, Yang W, Li H, Zhang B, Shuai Y, Chen Y (2015). Cardiovascular and all-cause Mortality over a 23-Year period among Chinese with newly diagnosed Diabetes in the Da Qing IGT and Diabetes Study. Diabetes Care.

[4] Tancredi M, Rosengren A, Svensson AM, Kosiborod M, Pivodic A, Gudbjörnsdottir S, Wedel H, Clements M, Dahlqvist S, Lind M (2015). Excess mortality among persons with type 2 Diabetes. N Engl J Med.

[5] Zaccardi F, Dhalwani NN, Papamargaritis D, Webb DR, Murphy GJ, Davies MJ, Khunti K (2017). Nonlinear association of BMI with all-cause and cardiovascular mortality in type 2 Diabetes Mellitus: a systematic review and meta-analysis of 414,587 participants in prospective studies. Diabetologia.

[6] Tate J, Knuiman M, Davis WA, Davis TME, Bruce DG (2020). A comparison of obesity indices in relation to mortality in type 2 Diabetes: the Fremantle Diabetes Study. Diabetologia.

[7] Dulloo AG, Jacquet J, Solinas G, Montani JP, Schutz Y (2010). Body composition phenotypes in pathways to obesity and the metabolic syndrome. Int J Obes (Lond).

[8] Sahakyan KR, Somers VK, Rodriguez-Escudero JP, Hodge DO, Carter RE, Sochor O, Coutinho T, Jensen MD, Roger VL, Singh P (2015). Normal-weight central obesity: implications for Total and Cardiovascular Mortality. Ann Intern Med.

[9] Sun Y, Liu B, Snetselaar LG, Wallace RB, Caan BJ, Rohan TE, Neuhouser ML, Shadyab AH, Chlebowski RT, Manson JE (2019). Association of Normal-Weight Central Obesity with all-cause and cause-Specific Mortality among Postmenopausal Women. JAMA Netw Open.

[10] Zhang C, Rexrode KM, van Dam RM, Li TY, Hu FB (2008). Abdominal obesity and the risk of all-cause, cardiovascular, and cancer mortality: sixteen years of follow-up in US women. Circulation.

[11] Krakauer NY, Krakauer JC (2012). A new body shape index predicts mortality hazard independently of body mass index. PLoS ONE.

[12] Ji M, Zhang S, An R (2018). Effectiveness of a body shape index (ABSI) in predicting chronic Diseases and mortality: a systematic review and meta-analysis. Obes Rev.

[13] Lee DY, Lee MY, Sung KC (2018). Prediction of mortality with a body shape index in Young asians: comparison with body Mass Index and Waist circumference. Obes (Silver Spring).

[14] Orsi E, Solini A, Penno G, Bonora E, Fondelli C, Trevisan R, Vedovato M, Cavalot F, Lamacchia O, Haxhi J (2022). Body mass index versus surrogate measures of central adiposity as Independent predictors of mortality in type 2 Diabetes. Cardiovasc Diabetol.

[15] MJ Health Research Foundation. MJ Health Database. [http://www.mjhrf.org/main/page/resource/en/#resource01].

[16] Wu X, Tsai SP, Tsao CK, Chiu ML, Tsai MK, Lu PJ, Lee JH, Chen CH, Wen C, Chang SS (2017). Cohort Profile: the Taiwan MJ Cohort: half a million Chinese with repeated health surveillance data. Int J Epidemiol.

[17] Committee ADAPP (2021). 2. Classification and diagnosis of Diabetes: standards of Medical Care in Diabetes—2022. Diabetes Care.

[18] Chang SS, Wen CP, Tsai MK, Lawlor DA, Yang YC, Gunnell D (2012). Adiposity, its related biologic risk factors, and Suicide: a cohort study of 542,088 Taiwanese adults. Am J Epidemiol.

[19] Yu T, Bo Y, Chang L-y, Liu X, Tam T, Lao XQ (2021). Adiposity and risk of death: a prospective cohort study of 463,002 adults. Clin Nutr.

[20] Hayes LD, Sculthorpe N, Herbert P, Baker JS, Spagna R, Grace FM (2015). Six weeks of conditioning exercise increases total, but not free testosterone in lifelong sedentary aging men. Aging Male.

[21] Jebb SA, Cole TJ, Doman D, Murgatroyd PR, Prentice AM (2000). Evaluation of the novel Tanita body-fat analyser to measure body composition by comparison with a four-compartment model. Br J Nutr.

[22] Abe T, Kearns CF, Fukunaga T (2003). Sex differences in whole body skeletal muscle mass measured by magnetic resonance imaging and its distribution in young Japanese adults. Br J Sports Med.

[23] Karastergiou K, Smith SR, Greenberg AS, Fried SK (2012). Sex differences in human adipose tissues - the biology of pear shape. Biol Sex Differ.

[24] Brozek J, Grande F, Anderson JT, Keys A (1963). DENSITOMETRIC ANALYSIS OF BODY COMPOSITION: REVISION OF SOME QUANTITATIVE ASSUMPTIONS. Ann N Y Acad Sci.

[25] Powell-Wiley TM, Poirier P, Burke LE, Després JP, Gordon-Larsen P, Lavie CJ, Lear SA, Ndumele CE, Neeland IJ, Sanders P (2021). Obesity and Cardiovascular Disease: A Scientific Statement from the American Heart Association. Circulation.

[26] Hughes RA, Heron J, Sterne JAC, Tilling K (2019). Accounting for missing data in statistical analyses: multiple imputation is not always the answer. Int J Epidemiol.

[27] Elze MC, Gregson J, Baber U, Williamson E, Sartori S, Mehran R, Nichols M, Stone GW, Pocock SJ (2017). Comparison of Propensity score methods and Covariate Adjustment: evaluation in 4 Cardiovascular studies. J Am Coll Cardiol.

[28] Cook NR, Ridker PM (2009). Advances in measuring the effect of individual predictors of cardiovascular risk: the role of reclassification measures. Ann Intern Med.

[29] Sun X, Cao L, Liu Y, Huang W, Pei C, Wang X, Feng S, Song B (2023). Sex- and age-specific differences in associations of a body shape index with all-cause and cardiovascular death risks among US adults with Diabetes. Nutr Metab Cardiovasc Dis.

[30] Carnethon MR, Rasmussen-Torvik LJ, Palaniappan L. The obesity Paradox in Diabetes. Curr Cardiol Rep 2014; 16. 10.1007/s11886-013-0446-3 24408674

[31] Ades PA, Savage PD. The obesity paradox: perception vs knowledge. *Mayo Clin Proc* 2010; 85:112–114. 10.4065/mcp.2009.0777 PMC2813817 20118385

[32] Banack HR, Stokes A (2017). The ‘obesity paradox’ may not be a paradox at all. Int J Obes (Lond).

[33] Finelli C, Sommella L, Gioia S, La Sala N, Tarantino G (2013). Should visceral fat be reduced to increase longevity?. Ageing Res Rev.

[34] Jayedi A, Khan TA, Aune D, Emadi A, Shab-Bidar S (2022). Body fat and risk of all-cause mortality: a systematic review and dose-response meta-analysis of prospective cohort studies. Int J Obes (Lond).

[35] Ritz P, Salle A, Audran M, Rohmer V (2007). Comparison of different methods to assess body composition of weight loss in obese and diabetic patients. Diabetes Res Clin Pract.

[36] Brizzolara A, Barbieri MP, Adezati L, Viviani GL (1996). Water distribution in insulin-dependent Diabetes Mellitus in various states of metabolic control. Eur J Endocrinol.

[37] Tsui EY, Gao XJ, Zinman B (1998). Bioelectrical impedance analysis (BIA) using bipolar foot electrodes in the assessment of body composition in type 2 Diabetes Mellitus. Diabet Med.

[38] Miyatani M, Yang P, Thomas S, Craven BC, Oh P. Bioelectrical Impedance and Dual-Energy X-Ray Absorptiometry Assessments of Changes in Body Composition Following Exercise in Patients with Type 2 Diabetes Mellitus. *Journal of Obesity* 2012; 2012:1–9. 10.1155/2012/953060 PMC3457637 23029604

[39] Kim S, Jeong JC, Ahn SY, Doh K, Jin DC, Na KY (2019). Time-varying effects of body mass index on mortality among hemodialysis patients: results from a nationwide Korean registry. Kidney Res Clin Pract.

[40] Zhu J, Liu X, Zhang J, Li J, Chen L, Huang C, Li J, Yu Y, Xu H, Qin G (2022). Time-varying association between body mass index and all-cause mortality in patients with Hypertension. Int J Obes (Lond).

[41] Sheen YJ, Hsu CC, Jiang YD, Huang CN, Liu JS, Sheu WH (2019). Trends in prevalence and incidence of Diabetes Mellitus from 2005 to 2014 in Taiwan. J Formos Med Assoc.

