# Supplementary material for: Obesity indices and the risk of total and cardiovascular mortality among people with diabetes: a long-term follow-up study in Taiwan
Source: Cardiovasc Diabetol. 2023 Dec 13;22:345. doi: 10.1186/s12933-023-02072-3 (PMC10720223; doi:10.1186/s12933-023-02072-3)

| Table S1. Diagnosis codes for death from cardiovascular diseases | | |
| --- | --- | --- |
|  | ICD-9-CM | ICD-10-CM |
| All-cause | 001–969 | A00–Z99 |
| Heart diseases  (Excluding hypertensive disease) | 390-392、393-398、410-414、420-429 | I01-I02.0、I05-I09、I20-I25、I27、I30-I52 |
| Hypertension diseases | 401-405 | I10-I15 |
| Cerebrovascular diseases | 430-438 | I60-I69 |
| Arteriolosclerosis | 440 | I70 |
| Aortic aneurysm and dissection |  | I71 |

Abbreviation: ICD-9-CM, International Classification of Diseases, Ninth Revision, Clinical Modification.

Table S2. Descriptive statistics of obesity indices in the study cohort

|  | Mean | SD | P20 | P40 | Median | P60 | P80 |
| --- | --- | --- | --- | --- | --- | --- | --- |
| Overall |  |  |  |  |  |  |  |
| BMI, kg/m^2^ | 25.90 | 4.02 | 22.70 | 24.63 | 25.52 | 26.44 | 28.83 |
| WC, cm | 86.07 | 10.41 | 77.00 | 83.00 | 86.00 | 88.00 | 94.00 |
| WHR | 0.88 | 0.07 | 0.82 | 0.87 | 0.89 | 0.90 | 0.95 |
| BF % | 29.64 | 8.47 | 22.60 | 26.80 | 28.80 | 30.90 | 36.50 |
| ABSI, m^11/6^ kg^−2/3^ | 0.077 | 0.005 | 0.073 | 0.076 | 0.077 | 0.078 | 0.081 |

Abbreviations: ABSI, A Body Shape Index; BF%, body fat percentage; BMI, body mass index; P20, 20^th^ percentile; P40, 40^th^ percentile; P60, 60^th^ percentile; P80, 80^th^ percentile; SD, Standard Deviation; WC, waist circumference; WHR, waist–hip ratio

| Table S3. Hazard ratios of cause-specific cardiovascular mortality in association with adiposity variables among people with diabetes | | | | | | | |
| --- | --- | --- | --- | --- | --- | --- | --- |
|  | Death from heart disease | | |  | Death from cerebrovascular disease | | |
|  | No. of deaths | Mortality^*^ | HR (95% CI) |  | No. of deaths | Mortality^*^ | HR (95% CI) |
| BMI, kg/m^2^ |  |  |  |  |  |  |  |
| 1^st^ quintile | 223 | 2.17 | 1.14 (0.89, 1.46) |  | 145 | 1.41 | 1.31 (0.97, 1.76) |
| 2^nd^ quintile | 217 | 2.11 | 0.99 (0.78, 1.27) |  | 122 | 1.19 | 0.81 (0.60, 1.11) |
| 3^rd^ quintile | 190 | 1.83 | 1.00 (Ref.) |  | 126 | 1.21 | 1.00 (Ref.) |
| 4^th^ quintile | 187 | 1.85 | 1.17 (0.91, 1.49) |  | 95 | 0.94 | 0.76 (0.55, 1.07) |
| 5^th^ quintile | 172 | 1.76 | 1.22 (0.94, 1.59) |  | 68 | 0.70 | 0.60 (0.40, 0.89) |
| Waist circumference, cm |  |  |  |  |  |  |  |
| 1^st^ quintile | 122 | 1.33 | 0.81 (0.60, 1.09) |  | 77 | 0.84 | 1.02 (0.72, 1.47) |
| 2^nd^ quintile | 199 | 1.94 | 0.95 (0.74, 1.23) |  | 109 | 1.06 | 0.88 (0.64, 1.21) |
| 3^rd^ quintile | 189 | 1.86 | 1.00 (Ref.) |  | 126 | 1.24 | 1.00 (Ref.) |
| 4^th^ quintile | 218 | 2.09 | 1.06 (0.83, 1.35) |  | 108 | 1.03 | 0.74 (0.54, 1.02) |
| 5^th^ quintile | 261 | 2.44 | 1.23 (0.97, 1.56) |  | 136 | 1.27 | 0.84 (0.61, 1.16) |
| Waist-hip ratio |  |  |  |  |  |  |  |
| 1^st^ quintile | 87 | 0.84 | 0.60 (0.43, 0.85) |  | 52 | 0.50 | 0.69 (0.43, 1.10) |
| 2^nd^ quintile | 191 | 1.69 | 0.80 (0.61, 1.05) |  | 107 | 0.95 | 1.06 (0.74, 1.52) |
| 3^rd^ quintile | 163 | 2.01 | 1.00 (Ref.) |  | 84 | 1.03 | 1.00 (Ref.) |
| 4^th^ quintile | 289 | 2.33 | 1.18 (0.93, 1.51) |  | 170 | 1.37 | 1.24 (0.89, 1.73) |
| 5^th^ quintile | 259 | 3.01 | 1.15 (0.89, 1.49) |  | 143 | 1.66 | 1.02 (0.71, 1.46) |
| Body fat, % |  |  |  |  |  |  |  |
| 1^st^ quintile | 294 | 2.98 | 1.48 (1.14, 1.93) |  | 165 | 1.67 | 1.33 (0.94, 1.87) |
| 2^nd^ quintile | 204 | 1.92 | 1.34 (1.02, 1.74) |  | 146 | 1.38 | 1.46 (1.05, 2.03) |
| 3^rd^ quintile | 159 | 1.58 | 1.00 (Ref.) |  | 91 | 0.90 | 1.00 (Ref.) |
| 4^th^ quintile | 172 | 1.70 | 1.14 (0.86, 1.51) |  | 85 | 0.84 | 0.82 (0.57, 1.19) |
| 5^th^ quintile | 160 | 1.58 | 1.05 (0.77, 1.42) |  | 69 | 0.68 | 0.60 (0.39, 0.91) |
| ABSI, m^11/6^ kg^−2/3^ |  |  |  |  |  |  |  |
| 1^st^ quintile | 73 | 0.81 | 0.74 (0.52, 1.05) |  | 28 | 0.31 | 0.51 (0.29, 0.89) |
| 2^nd^ quintile | 124 | 1.12 | 0.64 (0.47, 0.87) |  | 64 | 0.58 | 0.67 (0.44, 1.03) |
| 3^rd^ quintile | 159 | 1.78 | 1.00 (Ref.) |  | 72 | 0.81 | 1.00 (Ref.) |
| 4^th^ quintile | 223 | 2.01 | 0.81 (0.63, 1.05) |  | 141 | 1.27 | 1.18 (0.84, 1.66) |
| 5^th^ quintile | 410 | 3.84 | 1.17 (0.92, 1.48) |  | 251 | 2.35 | 1.29 (0.93, 1.80) |
| Abbreviations: ABSI, A Body Shape Index; BMI, body mass index; CI, confidence interval; HR, hazard ratio.  ^*^Per 1000 person-years  The model was adjusted for age, sex, marital status, education, smoking, alcohol drinking, leisure time physical activity, anti-hyperglycemic drugs, fasting glucose, hypertension, uric acid, cardiovascular disease (including stroke), chronic kidney disease, and cancer (n=24,257). | | | | | | | |

| Table S4. Distribution of missing values by quintile of A Body Shape Index (ABSI) |
| --- |

| Variables | All | A Body Shape Index (ABSI), m^11/6^ kg^−2/3^ (n= 34,686) | | | | |
| --- | --- | --- | --- | --- | --- | --- |
|  |  | <0.073 | 0.073 to <0.076 | 0.076 to <0.078 | 0.078 to <0.081 | ≥0.081 |
|  | (n=34,686) | (n= 5,959) | (n= 7,463) | (n= 6,105) | (n= 7,629) | (n= 7,530) |
|  | n (%) | n (%) | n (%) | n (%) | n (%) | n (%) |
| Sex | 0 (0.00) | 0 (0.00) | 0 (0.00) | 0 (0.00) | 0 (0.00) | 0 (0.00) |
| Age | 0 (0.00) | 0 (0.00) | 0 (0.00) | 0 (0.00) | 0 (0.00) | 0 (0.00) |
| Marital status | 2620 (7.55) | 447 (7.50) | 586 (7.85) | 473 (7.75) | 580 (7.60) | 534 (7.09) |
| Education | 1791 (5.16) | 303 (5.08) | 373 (5.00) | 304 (4.98) | 396 (5.19) | 415 (5.51) |
| Smoking | 2004 (5.78) | 302 (5.07) | 443 (5.94) | 343 (5.62) | 439 (5.75) | 477 (6.33) |
| Alcohol drinking | 3248 (9.36) | 575 (9.65) | 722 (9.67) | 535 (8.76) | 713 (9.35) | 703 (9.34) |
| Leisure time physical activity | 7029 (20.26) | 978 (16.41) | 1365 (18.29) | 1175 (19.25) | 1576 (20.66) | 1935 (25.70) |
| Chronic kidney disease | 11 (0.03) | 2 (0.03) | 3 (0.04) | 1 (0.02) | 4 (0.05) | 1 (0.01) |
| Hypertension | 104 (0.30) | 23 (0.39) | 27 (0.36) | 14 (0.23) | 28 (0.37) | 12 (0.16) |
| Anti-hyperglycemic drugs | 123 (0.35) | 25 (0.42) | 32 (0.43) | 20 (0.33) | 29 (0.38) | 17 (0.23) |
| Cancer | 90 (0.26) | 21 (0.35) | 25 (0.33) | 14 (0.23) | 19 (0.25) | 11 (0.15) |
| Cardiovascular disease | 567 (1.63) | 103 (1.73) | 130 (1.74) | 113 (1.85) | 138 (1.81) | 83 (1.10) |
| Stroke | 90 (0.26) | 21 (0.35) | 25 (0.33) | 14 (0.23) | 19 (0.25) | 11 (0.15) |
| Uric acid | 223 (0.64) | 18 (0.30) | 47 (0.63) | 46 (0.75) | 63 (0.83) | 49 (0.65) |
| Triglycerides | 12 (0.03) | 3 (0.05) | 3 (0.04) | 1 (0.02) | 3 (0.04) | 2 (0.03) |
| Total cholesterol | 10 (0.03) | 3 (0.05) | 2 (0.03) | 2 (0.03) | 1 (0.01) | 2 (0.03) |
| Fasting glucose | 7 (0.02) | 1 (0.02) | 2 (0.03) | 2 (0.03) | 1 (0.01) | 1 (0.01) |

| Table S5. Hazard ratios (95% confidence interval) of all-cause mortality in association with adiposity variables among people with diabetes in the multiple imputation data set | | |
| --- | --- | --- |
|  | Model 1 | Model 2 |
| BMI, kg/m^2^ |  |  |
| 1^st^ quintile | 1.32 (1.24, 1.41) | 1.35 (1.26, 1.44) |
| 2^nd^ quintile | 1.09 (1.02, 1.16) | 1.09 (1.02, 1.16) |
| 3^rd^ quintile | 1.00 (Ref.) | 1.00 (Ref.) |
| 4^th^ quintile | 1.08 (1.01, 1.16) | 1.06 (0.99, 1.14) |
| 5^th^ quintile | 1.16 (1.08, 1.25) | 1.11 (1.03, 1.20) |
| Waist circumference, cm |  |  |
| 1^st^ quintile | 1.03 (0.96, 1.11) | 1.10 (1.02, 1.19) |
| 2^nd^ quintile | 0.93 (0.87, 0.99) | 0.95 (0.88, 1.01) |
| 3^rd^ quintile | 1.00 (Ref.) | 1.00 (Ref.) |
| 4^th^ quintile | 0.95 (0.89, 1.02) | 0.95 (0.89, 1.01) |
| 5^th^ quintile | 1.12 (1.04, 1.19) | 1.08 (1.01, 1.15) |
| Waist-hip ratio |  |  |
| 1^st^ quintile | 0.83 (0.76, 0.91) | 0.89 (0.82, 0.98) |
| 2^nd^ quintile | 0.96 (0.90, 1.04) | 0.97 (0.90, 1.05) |
| 3^rd^ quintile | 1.00 (Ref.) | 1.00 (Ref.) |
| 4^th^ quintile | 1.10 (1.03, 1.18) | 1.07 (0.99, 1.14) |
| 5^th^ quintile | 1.14 (1.06, 1.22) | 1.06 (0.99, 1.14) |
| Body fat, % |  |  |
| 1^st^ quintile | 1.43 (1.33, 1.54) | 1.48 (1.38, 1.59) |
| 2^nd^ quintile | 1.14 (1.06, 1.23) | 1.18 (1.09, 1.26) |
| 3^rd^ quintile | 1.00 (Ref.) | 1.00 (Ref.) |
| 4^th^ quintile | 0.98 (0.90, 1.05) | 0.97 (0.90, 1.05) |
| 5^th^ quintile | 0.95 (0.87, 1.03) | 0.91 (0.84, 0.98) |
| ABSI, m^11/6^ kg^−2/3^ |  |  |
| 1^st^ quintile | 0.76 (0.69, 0.84) | 0.80 (0.73, 0.89) |
| 2^nd^ quintile | 0.91 (0.84, 0.99) | 0.93 (0.86, 1.01) |
| 3^rd^ quintile | 1.00 (Ref.) | 1.00 (Ref.) |
| 4^th^ quintile | 1.09 (1.01, 1.17) | 1.07 (0.99, 1.15) |
| 5^th^ quintile | 1.32 (1.23, 1.41) | 1.27 (1.19, 1.36) |
| Abbreviations: ABSI, A Body Shape Index; BMI, body mass index.  Model 1 was adjusted for age, sex, marital status, education, smoking, alcohol, physical activity, and anti-hyperglycemic drugs; Model 2 included Model 1 variables plus fasting glucose, hypertension, uric acid, cardiovascular disease (including stroke), chronic kidney disease, and cancer. | | |

| Table S6. Hazard ratios (95% confidence interval) of cardiovascular mortality in association with adiposity variables among people with diabetes in the multiple imputation data set | | |
| --- | --- | --- |
|  | Model 1 | Model 2 |
| BMI, kg/m^2^ |  |  |
| 1^st^ quintile | 1.18 (1.02, 1.36) | 1.29 (1.12, 1.49) |
| 2^nd^ quintile | 1.01 (0.87, 1.16) | 1.03 (0.89, 1.19) |
| 3^rd^ quintile | 1.00 (Ref.) | 1.00 (Ref.) |
| 4^th^ quintile | 1.04 (0.89, 1.20) | 0.99 (0.86, 1.16) |
| 5^th^ quintile | 1.23 (1.05, 1.44) | 1.10 (0.94, 1.29) |
| Waist circumference, cm |  |  |
| 1^st^ quintile | 0.93 (0.78, 1.10) | 1.05 (0.88, 1.25) |
| 2^nd^ quintile | 0.95 (0.82, 1.11) | 0.99 (0.85, 1.15) |
| 3^rd^ quintile | 1.00 (Ref.) | 1.00 (Ref.) |
| 4^th^ quintile | 0.95 (0.82, 1.10) | 0.93 (0.80, 1.07) |
| 5^th^ quintile | 1.27 (1.11, 1.46) | 1.14 (0.99, 1.32) |
| Waist-hip ratio |  |  |
| 1^st^ quintile | 0.64 (0.53, 0.79) | 0.71 (0.58, 0.87) |
| 2^nd^ quintile | 0.91 (0.77, 1.07) | 0.92 (0.78, 1.08) |
| 3^rd^ quintile | 1.00 (Ref.) | 1.00 (Ref.) |
| 4^th^ quintile | 1.20 (1.03, 1.39) | 1.13 (0.97, 1.31) |
| 5^th^ quintile | 1.20 (1.03, 1.40) | 1.05 (0.90, 1.22) |
| Body fat, % |  |  |
| 1^st^ quintile | 1.30 (1.11, 1.52) | 1.42 (1.21, 1.66) |
| 2^nd^ quintile | 1.21 (1.04, 1.42) | 1.28 (1.10, 1.50) |
| 3^rd^ quintile | 1.00 (Ref.) | 1.00 (Ref.) |
| 4^th^ quintile | 1.05 (0.89, 1.25) | 1.03 (0.87, 1.22) |
| 5^th^ quintile | 0.99 (0.83, 1.19) | 0.89 (0.75, 1.07) |
| ABSI, m^11/6^ kg^−2/3^ |  |  |
| 1^st^ quintile | 0.73 (0.58, 0.91) | 0.77 (0.62, 0.96) |
| 2^nd^ quintile | 0.80 (0.66, 0.95) | 0.81 (0.67, 0.97) |
| 3^rd^ quintile | 1.00 (Ref.) | 1.00 (Ref.) |
| 4^th^ quintile | 1.06 (0.90, 1.24) | 1.03 (0.88, 1.20) |
| 5^th^ quintile | 1.32 (1.14, 1.53) | 1.25 (1.08, 1.45) |
| Abbreviations: ABSI, A Body Shape Index; BMI, body mass index.  Model 1 was adjusted for age, sex, marital status, education, smoking, alcohol, physical activity, and anti-hyperglycemic drugs; Model 2 included Model 1 variables plus fasting glucose, hypertension, uric acid, cardiovascular disease (including stroke), chronic kidney disease, and cancer. | | |

| Table S7. Baseline characteristics of subjects with diabetes in the propensity score matched-pair sample (ABSI) | | | |
| --- | --- | --- | --- |
|  | ABSI, m^11/6^ kg^−2/3^ | |  |
|  | <5^th^ quintile (< 0.081) | ≥ 5^th^ quintile (≥ 0.081) | P value |
| Number of subjects | 4551 | 4551 |  |
| Sex, n (%) |  |  | 0.89 |
| Men | 3216 (70.67) | 3222 (70.80) |  |
| Women | 1335 (29.33) | 1329 (29.20) |  |
| Age, mean (SD), years | 60.48 (10.56) | 60.47 (10.72) | 0.97 |
| 18-44 | 376 (8.26) | 378 (8.31) | 0.89 |
| 45-64 | 2485 (54.60) | 2462 (54.10) |  |
| ≥65 | 1690 (37.13) | 1711 (37.60) |  |
| Marital status, n (%) |  |  | 0.90 |
| Single | 120 (2.64) | 113 (2.48) |  |
| Married | 3698 (81.26) | 3704 (81.39) |  |
| Widowed/Divorce | 733 (16.11) | 734 (16.13) |  |
| Education, n (%) |  |  | 0.78 |
| Illiterate | 579 (12.72) | 567 (12.46) |  |
| Junior secondary school or below | 1942 (42.67) | 1925 (42.30) |  |
| Senior secondary school | 791 (17.38) | 828 (18.19) |  |
| College or above | 1239 (27.22) | 1231 (27.05) |  |
| Smoking, n (%) |  |  | 0.94 |
| Never | 2677 (58.82) | 2661 (58.47) |  |
| Quit | 633 (13.91) | 639 (14.04) |  |
| Current | 1241 (27.27) | 1251 (27.49) |  |
| Alcohol drinking, n (%) |  |  | 0.39 |
| Never | 3126 (68.69) | 3080 (67.68) |  |
| Quit | 315 (6.92) | 346 (7.60) |  |
| Current | 1110 (24.39) | 1125 (24.72) |  |
| Leisure time physical activity, n (%) |  |  | 0.96 |
| Sedentary | 85 (1.87) | 90 (1.98) |  |
| Light activity | 3610 (79.32) | 3615 (79.43) |  |
| Moderate activity | 508 (11.16) | 496 (10.90) |  |
| Vigorous activity | 348 (7.65) | 350 (7.69) |  |
| Chronic kidney disease, n (%) | 802 (17.62) | 804 (17.67) | 0.96 |
| Hypertension, n (%) | 2007 (44.10) | 1972 (43.33) | 0.46 |
| Anti-hyperglycemic drugs, n (%) | 2130 (46.80) | 2134 (46.89) | 0.93 |
| Cancer, n (%) | 131 (2.88) | 145 (3.19) | 0.39 |
| Cardiovascular disease, n (%) | 597 (13.31) | 578 (12.83) | 0.51 |
| Stroke, n (%) | 101 (2.22) | 123 (2.70) | 0.14 |
| Uric acid, mg/dL | 6.24 (1.63) | 6.28 (1.66) | 0.32 |
| Fasting glucose, mg/dL | 161.7 (54.97) | 161.8 (54.65) | 0.89 |
| Abbreviation: ABSI, A Body Shape Index | | | |

| Table S8. Hazard ratios of all-cause and cardiovascular mortality in association with ABSI in the original cohort and propensity score matched-pair sample | | | | | | | |
| --- | --- | --- | --- | --- | --- | --- | --- |
|  | Original cohort^*^ | | |  | Propensity score matched-pair sample | | |
|  | No. of participants | No. of deaths | HR (95% CI) |  | No. of participants | No. of deaths | HR (95% CI) |
| All-cause mortality |  |  |  |  |  |  |  |
| ABSI, m^11/6^ kg^−2/3^ |  |  |  |  |  |  |  |
| <5^th^ quintile | 217156 | 4942 | Ref. |  | 4551 | 1412 | Ref. |
| ≥ 5^th^ quintile | 7530 | 3382 | 1.27 (1.20-1.35) |  | 4551 | 1832 | 1.20 (1.12-1.28) |
| Cardiovascular mortality |  |  |  |  |  |  |  |
| ABSI, m^11/6^ kg^−2/3^ |  |  |  |  |  |  |  |
| <5^th^ quintile | 27156 | 1009 | Ref. |  | 4551 | 298 | Ref. |
| ≥ 5^th^ quintile | 7530 | 739 | 1.35 (1.19-1.54) |  | 4551 | 410 | 1.26 (1.09-1.46) |
| Abbreviations: ABSI, A Body Shape Index; CI, confidence interval; HR, Hazard ratio.  ^*^Model was adjusted for age, sex, marital status, education, smoking, alcohol drinking, leisure time physical activity, anti-hyperglycemic drugs, fasting glucose, hypertension, uric acid, cardiovascular disease (including stroke), chronic kidney disease, and cancer (n=24,257). | | | | | | | |

Table S9. Associations between A Body Shape Index (ABSI) and all-cause mortality in subgroups of age, sex, smoking, chronic kidney disease, cardiovascular disease, and BMI

|  | Number of participants | Number of deaths | Hazard ratio (95% confidence interval) (reference group: 3^rd^ quintile) | | | | P for trend | P for interaction |
| --- | --- | --- | --- | --- | --- | --- | --- | --- |
|  |  |  | 1st quintile | 2nd quintile | 4th quintile | 5th quintile |  |  |
| Age, years |  |  |  |  |  |  |  | 0.065 |
| 18-44 | 8262 | 474 | 0.51 (0.34-0.79) | 0.90 (0.64-1.25) | 0.71 (0.48-1.04) | 1.24 (0.81-1.88) | 0.009 |  |
| 45-64 | 19455 | 4027 | 0.79 (0.67-0.93) | 0.92 (0.80-1.06) | 1.16 (1.02-1.32) | 1.50 (1.32-1.70) | <0.0001 |  |
| ≥65 | 6969 | 3823 | 0.68 (0.54-0.85) | 0.83 (0.71-0.98) | 1.00 (0.87-1.14) | 1.22 (1.08-1.38) | <0.0001 |  |
| Sex |  |  |  |  |  |  |  | 0.010 |
| Women | 15171 | 3662 | 0.77 (0.66-0.90) | 0.86 (0.74-0.99) | 1.01 (0.88-1.16) | 1.36 (1.19-1.54) | <0.0001 |  |
| Men | 19515 | 4662 | 0.87 (0.68-1.11) | 1.01 (0.87-1.17) | 1.07 (0.95-1.20) | 1.19 (1.06-1.34) | <0.0001 |  |
| Smoking |  |  |  |  |  |  |  | 0.35 |
| Never | 21894 | 4903 | 0.81 (0.70-0.93) | 0.90 (0.80-1.02) | 1.04 (0.93-1.17) | 1.26 (1.14-1.41) | <0.0001 |  |
| Ever | 10788 | 2883 | 0.72 (0.53-0.99) | 0.99 (0.82-1.20) | 1.05 (0.90-1.23) | 1.24 (1.07-1.43) | <0.0001 |  |
| Chronic kidney disease |  |  |  |  |  |  |  | 0.60 |
| Yes | 4144 | 2151 | 0.72 (0.55-0.94) | 0.87 (0.70-1.08) | 1.00 (0.83-1.20) | 1.20 (1.01-1.42) | <0.0001 |  |
| No | 30531 | 6168 | 0.81 (0.71-0.94) | 0.93 (0.83-1.05) | 1.05 (0.95-1.17) | 1.25 (1.13-1.38) | <0.0001 |  |
| Cardiovascular disease |  |  |  |  |  |  |  | 0.302 |
| Yes | 3560 | 1577 | 0.90 (0.68-1.21) | 0.88 (0.69-1.14) | 1.09 (0.89-1.35) | 1.26 (1.03-1.53) | 0.0003 |  |
| No | 31036 | 6747 | 0.76 (0.67-0.88) | 0.92 (0.83-1.03) | 1.04 (0.94-1.15) | 1.25 (1.14-1.37) | <0.0001 |  |
| BMI, kg/m^2^ |  |  |  |  |  |  |  | 0.001 |
| 1^st^ quintile | 6945 | 2029 | 0.68 (0.52-0.89) | 0.81 (0.66-1.00) | 0.96 (0.80-1.15) | 1.21 (1.02-1.43) | <0.0001 |  |
| 2^nd^ quintile | 6922 | 1855 | 1.02 (0.78-1.34) | 1.07 (0.86-1.34) | 1.24 (1.02-1.51) | 1.35 (1.11-1.63) | 0.001 |  |
| 3^rd^ quintile | 6948 | 1636 | 0.75 (0.56-1.01) | 0.99 (0.78-1.26) | 1.40 (1.14-1.71) | 1.30 (1.07-1.60) | <0.0001 |  |
| 4^th^ quintile | 6930 | 1531 | 0.91 (0.69-1.19) | 0.97 (0.78-1.22) | 0.83 (0.67-1.02) | 1.14 (0.94-1.38) | 0.067 |  |
| 5^th^ quintile | 6941 | 1273 | 0.63 (0.47-0.84) | 0.80 (0.63-1.03) | 0.90 (0.71-1.14) | 1.39 (1.11-1.74) | <0.0001 |  |

Models were adjusted for age, sex, marital status, education, smoking, alcohol drinking, leisure time physical activity, anti-hyperglycemic drugs, fasting glucose, hypertension, uric acid, cardiovascular disease (including stroke), chronic kidney disease, and cancer.

Table S10. Associations between A Body Shape Index (ABSI) and cardiovascular mortality in subgroups of age, sex, smoking, chronic kidney disease, cardiovascular disease, and BMI

|  | Number of participants | Number of deaths | Hazard ratio (95% confidence interval) (reference group: 3^st^ quintile) | | | | P for trend | P for interaction |
| --- | --- | --- | --- | --- | --- | --- | --- | --- |
|  |  |  | 1st quintile | 2nd quintile | 4th quintile | 5th quintile |  |  |
| Age, years |  |  |  |  |  |  |  | 0.36 |
| 18-44 | 8262 | 106 | 0.44 (0.17-1.14) | 0.93 (0.46-1.89) | 0.77 (0.34-1.75) | 1.29 (0.54-3.12) | 0.12 |  |
| 45-64 | 19455 | 802 | 0.74 (0.51-1.08) | 0.72 (0.51-0.99) | 1.00 (0.75-1.35) | 1.49 (1.13-1.96) | <0.0001 |  |
| ≥65 | 6969 | 840 | 0.73 (0.46-1.14) | 0.55 (0.38-0.82) | 1.03 (0.78-1.37) | 1.21 (0.93-1.57) | <0.0001 |  |
| Sex |  |  |  |  |  |  |  | 0.37 |
| Women | 15171 | 753 | 0.69 (0.50-0.97) | 0.60 (0.43-0.82) | 0.95 (0.71-1.26) | 1.18 (0.90-1.54) | <0.0001 |  |
| Men | 19515 | 995 | 0.98 (0.59-1.64) | 0.87 (0.62-1.24) | 1.10 (0.84-1.43) | 1.34 (1.04-1.72) | 0.001 |  |
| Smoking |  |  |  |  |  |  |  | 0.36 |
| Never | 21894 | 1037 | 0.79 (0.58-1.06) | 0.68 (0.52-0.90) | 1.04 (0.82-1.33) | 1.33 (1.06-1.67) | <0.0001 |  |
| Ever | 10788 | 602 | 0.65 (0.32-1.33) | 0.80 (0.51-1.25) | 0.96 (0.69-1.34) | 1.10 (0.81-1.51) | 0.052 |  |
| Chronic kidney disease |  |  |  |  |  |  |  | 0.065 |
| Yes | 4144 | 490 | 0.68 (0.39-1.19) | 0.54 (0.32-0.90) | 1.23 (0.85-1.77) | 1.20 (0.84-1.71) | 0.001 |  |
| No | 30531 | 1257 | 0.77 (0.57-1.05) | 0.75 (0.57-0.97) | 0.91 (0.72-1.15) | 1.23 (0.99-1.53) | <0.0001 |  |
| Cardiovascular disease |  |  |  |  |  |  |  | 0.369 |
| Yes | 3560 | 446 | 0.56 (0.31-1.02) | 0.64 (0.40-1.03) | 0.96 (0.66-1.38) | 1.02 (0.71-1.45) | 0.020 |  |
| No | 31036 | 1302 | 0.82 (0.60-1.12) | 0.72 (0.55-0.95) | 1.04 (0.83-1.31) | 1.34 (1.08-1.66) | <0.0001 |  |
| BMI, kg/m^2^ |  |  |  |  |  |  |  | 0.683 |
| 1^st^ quintile | 6945 | 400 | 0.80 (0.44-1.44) | 0.47 (0.27-0.82) | 1.06 (0.70-1.60) | 1.17 (0.80-1.72) | 0.004 |  |
| 2^nd^ quintile | 6922 | 380 | 0.82 (0.43-1.55) | 0.89 (0.54-1.47) | 0.84 (0.54-1.30) | 1.18 (0.79-1.77) | 0.102 |  |
| 3^rd^ quintile | 6948 | 360 | 0.82 (0.46-1.47) | 0.71 (0.42-1.21) | 1.35 (0.88-2.04) | 1.24 (0.82-1.88) | 0.024 |  |
| 4^th^ quintile | 6930 | 319 | 0.81 (0.45-1.47) | 0.64 (0.38-1.09) | 0.84 (0.55-1.28) | 1.12 (0.76-1.67) | 0.072 |  |
| 5^th^ quintile | 6941 | 289 | 0.57 (0.29-1.11) | 0.90 (0.52-1.55) | 1.07 (0.64-1.78) | 1.78 (1.10-2.89) | <0.0001 |  |

Models were adjusted for age, sex, marital status, education, smoking, alcohol drinking, leisure time physical activity, anti-hyperglycemic drugs, fasting glucose, hypertension, uric acid, cardiovascular disease (including stroke), chronic kidney disease, and cancer.

Table S11. Associations between A Body Shape Index (ABSI) and all-cause and cardiovascular mortality stratified by weight change during follow-up period (n=14,223)

|  | No. of participants | No. of deaths | Hazard ratio (95% confidence interval) (reference group: 3^rd^ quintile) | | | | P for trend |
| --- | --- | --- | --- | --- | --- | --- | --- |
|  |  |  | 1st quintile | 2nd quintile | 4th quintile | 5th quintile |  |
| All-cause mortality |  |  |  |  |  |  |  |
| Weight change ≥5% | 3234 | 629 | 0.72 (0.47, 1.12) | 1.14 (0.80, 1.63) | 1.49 (1.07, 2.09) | 2.42 (1.75, 3.35) | <0.0001 |
| Weight change < 5% | 10989 | 1966 | 0.56 (0.44, 0.71) | 0.92 (0.76, 1.11) | 1.36 (1.14, 1.62) | 1.93 (1.62, 2.28) | <0.0001 |
| Cardiovascular mortality |  |  |  |  |  |  |  |
| Weight change ≥5% | 3234 | 129 | 0.80 (0.28, 2.31) | 1.70 (0.77, 3.77) | 1.83 (0.84, 4.00) | 2.56 (1.18, 5.55) | 0.013 |
| Weight change < 5% | 10989 | 397 | 0.59 (0.34, 1.04) | 0.88 (0.56, 1.39) | 1.62 (1.10, 2.41) | 1.94 (1.31, 2.87) | <0.0001 |

Models were adjusted for age, sex, marital status, education, smoking, alcohol drinking, leisure time physical activity, anti-hyperglycemic drugs, fasting glucose, hypertension, uric acid, cardiovascular disease (including stroke), chronic kidney disease, and cancer.

| Table S12. Performance of models containing different obesity measures in prediction of all-cause and cardiovascular mortality | | | | | | | | | |
| --- | --- | --- | --- | --- | --- | --- | --- | --- | --- |
|  | All-cause mortality | | |  | | Cardiovascular mortality | | | |
|  |  | | |  | |  | | | |
|  | C-statistic (95% CI) | NRI | IDI | |  | | C-statistic (95% CI) | NRI | IDI |
| Model 1^*^ | 0.747 (0.742-0.753) |  |  | |  | | 0.759 (0.747-0.772) |  |  |
| +BMI | 0.750 (0.744-0.755) | -0.006 | 0.0004 | |  | | 0.760 (0.749-0.773) | -0.028 | 0.0002 |
| +waist circumference | 0.748 (0.743-0.753) | -0.021 | 0.0002 | |  | | 0.762 (0.750-0.774) | 0.053 | 0.0006 |
| +waist-hip ratio | 0.750 (0.744-0.755) | 0.146 | 0.0012 | |  | | 0.765 (0.753-0.777) | 0.251 | 0.0012 |
| +body fat percentage | 0.752 (0.746-0.757) | 0.076 | 0.0013 | |  | | 0.762 (0.750-0.774) | 0.130 | 0.0004 |
| +ABSI | 0.754 (0.748-0.759) | 0.257 | 0.0044 | |  | | 0.767 (0.755-0.779) | 0.159 | 0.0019 |
| Model 3^*^ | 0.785 (0.779-0.792) |  |  | |  | | 0.811 (0.797-0.825) |  |  |
| +BMI | 0.787 (0.780-0.793) | -0.043 | 0.0003 | |  | | 0.812 (0.798-0.825) | -0.028 | 0.0002 |
| +waist circumference | 0.785 (0.780-0.792) | -0.057 | 0.0001 | |  | | 0.812 (0.799-0.826) | 0.036 | 0.0004 |
| +waist-hip ratio | 0.785 (0.780-0.792) | 0.174 | 0.0004 | |  | | 0.814 (0.801-0.827) | 0.245 | 0.0007 |
| +body fat percentage | 0.788 (0.781-0.794) | 0.080 | 0.0011 | |  | | 0.813 (0.799-0.826) | 0.220 | 0.0006 |
| +ABSI | 0.787 (0.781-0.794) | 0.173 | 0.0016 | |  | | 0.814 (0.800-0.828) | 0.184 | 0.0012 |
| Abbreviations: ABSI, A Body Shape Index; CI, confidence interval; IDI, integrated discrimination improvement; NRI, continuous net reclassification improvement.  ^*^Model 1 was adjusted for age and sex; model 3 was additionally adjusted for marital status, education, smoking, alcohol drinking, leisure time physical activity, anti-hyperglycemic drugs, fasting glucose, hypertension, uric acid, cardiovascular disease (including stroke), chronic kidney disease, and cancer (n=24,257). | | | | | | | | | |

Figure S1. Flowchart of selection of study participants.

Abbreviations: ABSI, A Body Shape Index; BFP, body fat percentage; BMI, body mass index; WHR, waist-hip ratio.

**Exclusions:**

1. Missing data for BMI, waist circumference, WHR, BFP, or ABSI at baseline (i.e., the date of health check-up where the criteria of diabetes diagnosis was met) (n= 3,978)
2. Age of < 18 years at baseline (n= 91)
3. Follow-up time less than one year (n= 178)

Participants with diabetes (n= 38,933)

Participants who had received health check-up at the MJ Health Management Institution from 1996 to 2017 (n= 615,353)

Participants included in the data analysis (n= 34,686)

Figure S2. Exposure–response relationship between the obesity indicators and all-cause mortality.

The curves (solid line) represent adjusted hazard ratios (HRs) based on restricted cubic splines with knots at the the 5th, 35th, 65th and 95th percentiles of distribution of each obesity measure. The gray shaded areas represent 95% confidence intervals (CIs). The reference values were set at the 50th percentile. White bars indicate the frequency distribution of the obesity indices. The models were adjusted for age, sex, marital status, education, smoking, alcohol, physical activity, anti-hyperglycemic drugs, fasting glucose, hypertension, uric acid, cardiovascular disease (including stroke), chronic kidney disease, and cancer. Abbreviations: ABSI, A Body Shape Index; BMI, body mass index; WHR, waist-hip ratio.


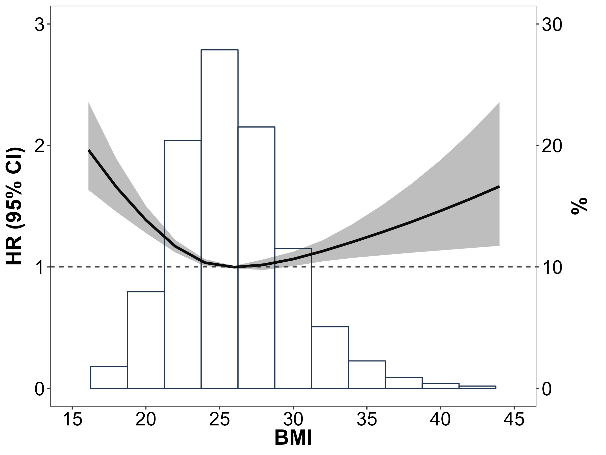

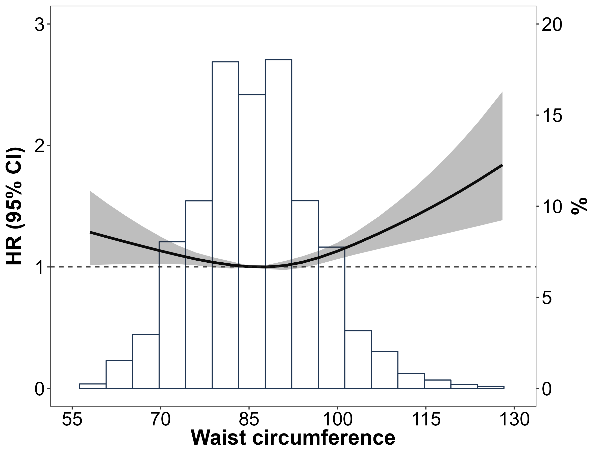

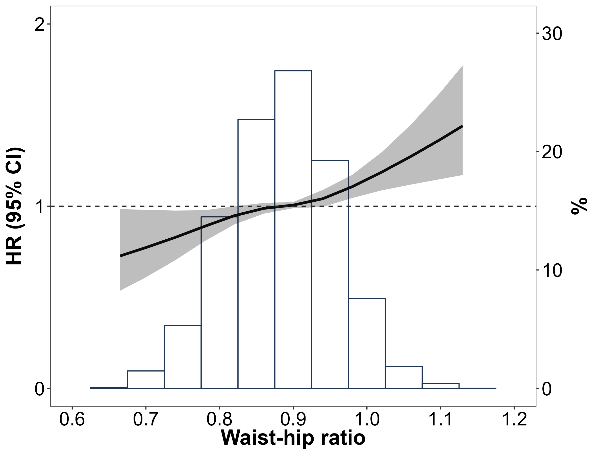

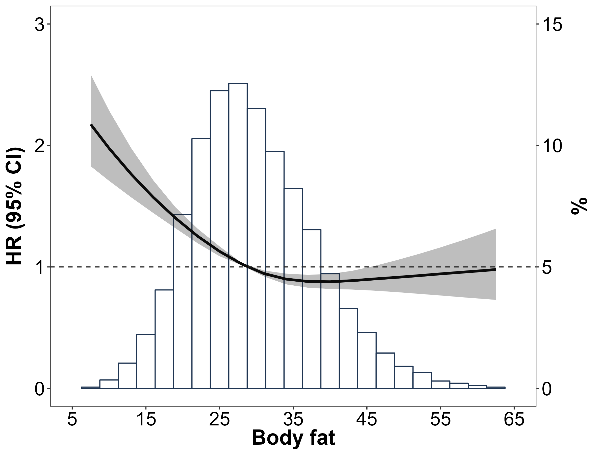


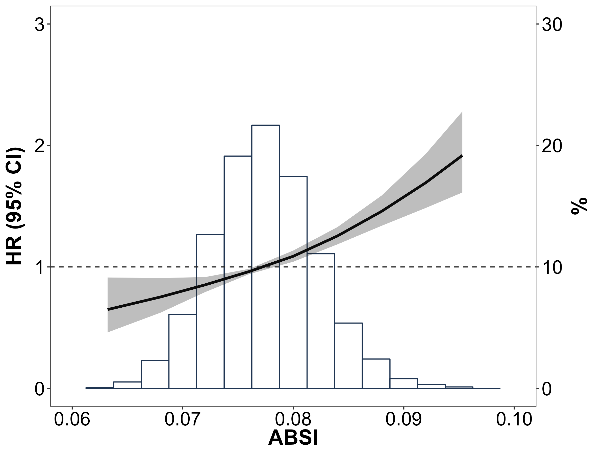


Figure S3. Exposure–response relationship between the obesity indicators and cardiovascular mortality.

The curves (solid line) represent adjusted hazard ratios (HRs) based on restricted cubic splines with knots at the the 5th, 35th, 65th and 95th percentiles of distribution of each obesity measure. The gray shaded areas represent 95% confidence intervals (CIs). The reference values were set at the 50th percentile. White bars indicate the frequency distribution of the obesity indices. The models were adjusted for age, sex, marital status, education, smoking, alcohol, physical activity, anti-hyperglycemic drugs, fasting glucose, hypertension, uric acid, cardiovascular disease (including stroke), chronic kidney disease, and cancer. Abbreviations: ABSI, A Body Shape Index; BMI, body mass index; WHR, waist-hip ratio.


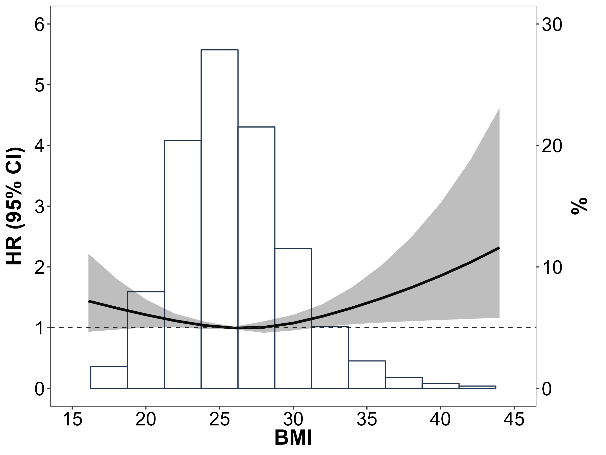

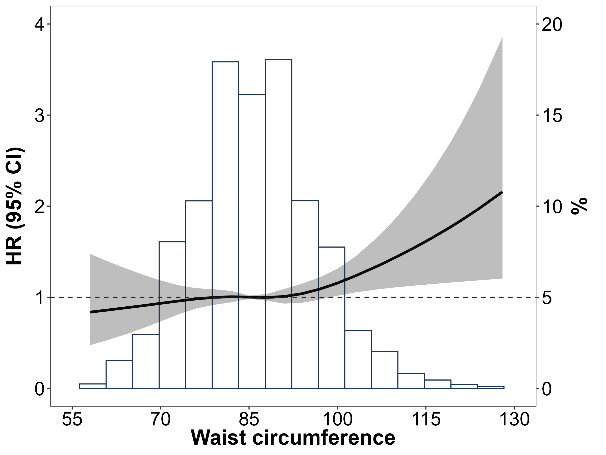

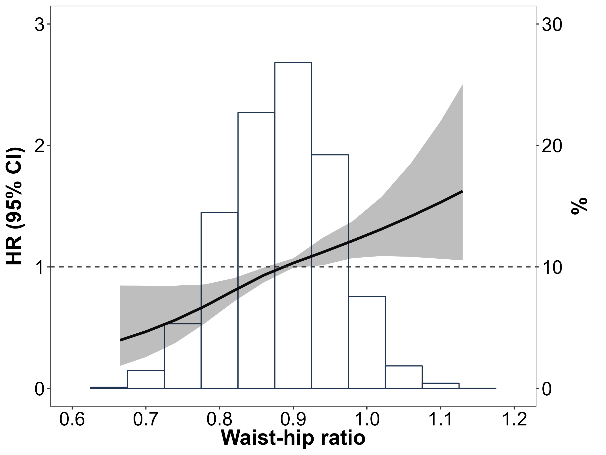

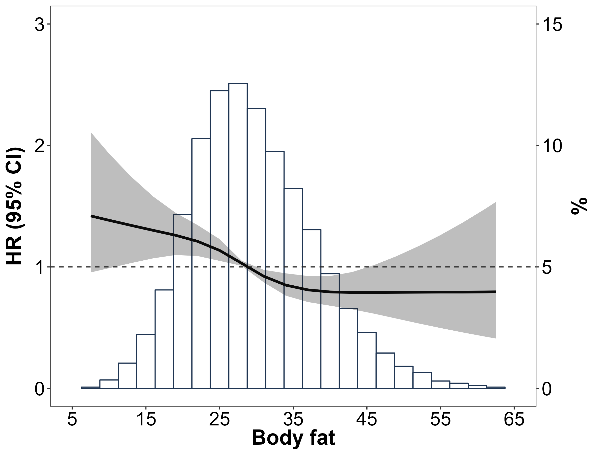


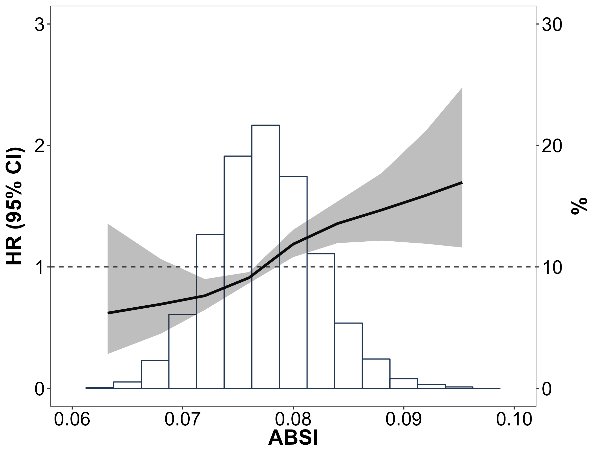


Figure S4. Hazard ratios (HR) for mortality in association with BMI stratified by follow-up duration. Models were adjusted for age, sex, marital status, education, smoking, alcohol drinking, leisure time physical activity, anti-hyperglycemic drugs, fasting glucose, hypertension, uric acid, cardiovascular disease (including stroke), chronic kidney disease, and cancer. Abbreviations: BMI, body mass index; CI, confidence interval

1. All-cause mortality


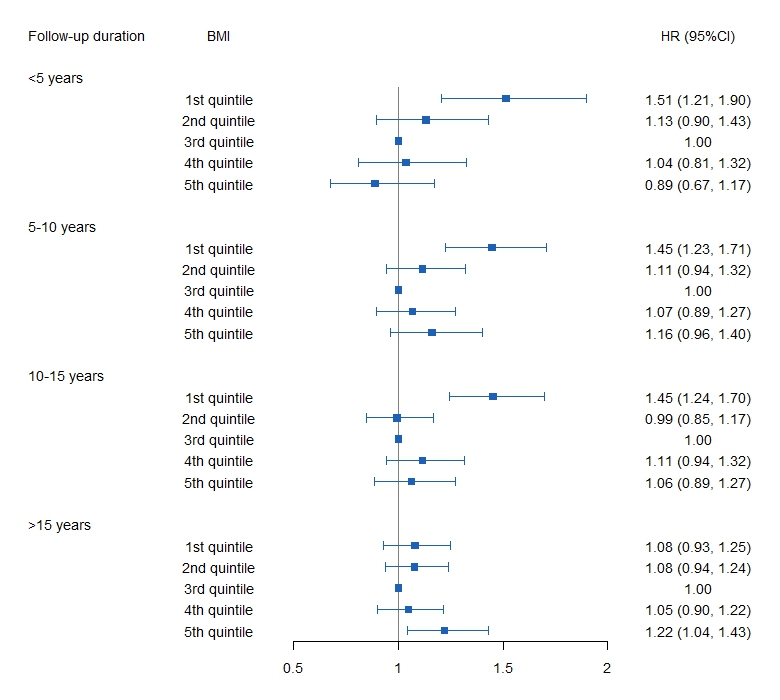


1. Cardiovascular mortality


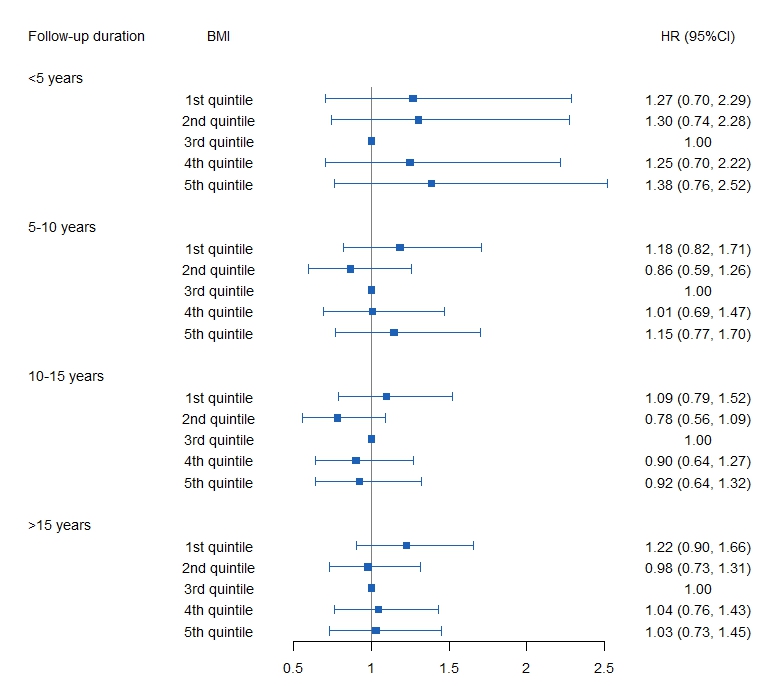


Figure S5. Hazard ratios (HR) for mortality in association with WC stratified by follow-up duration. Models were adjusted for age, sex, marital status, education, smoking, alcohol drinking, leisure time physical activity, anti-hyperglycemic drugs, fasting glucose, hypertension, uric acid, cardiovascular disease (including stroke), chronic kidney disease, and cancer. Abbreviations: WC, waist circumference; CI, confidence interval.

1. All-cause mortality


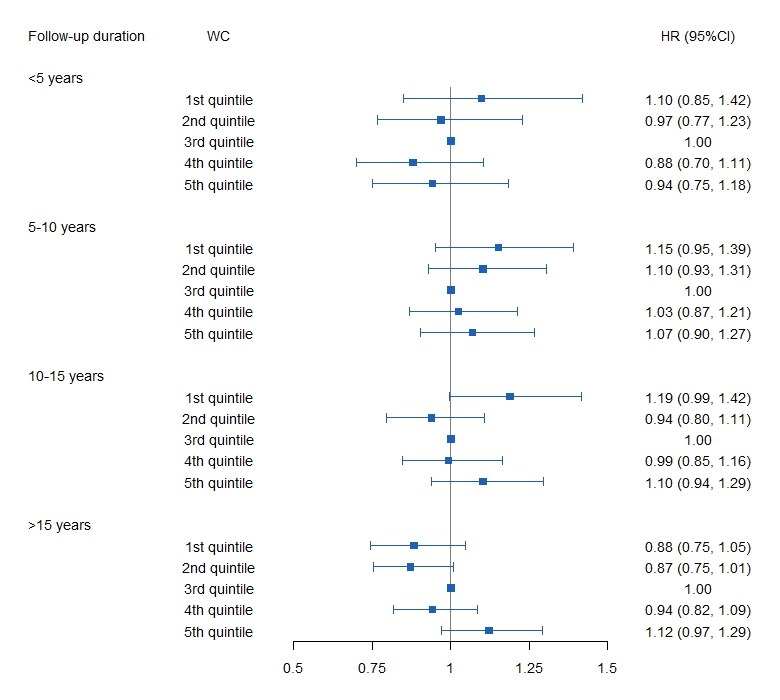


1. Cardiovascular mortality


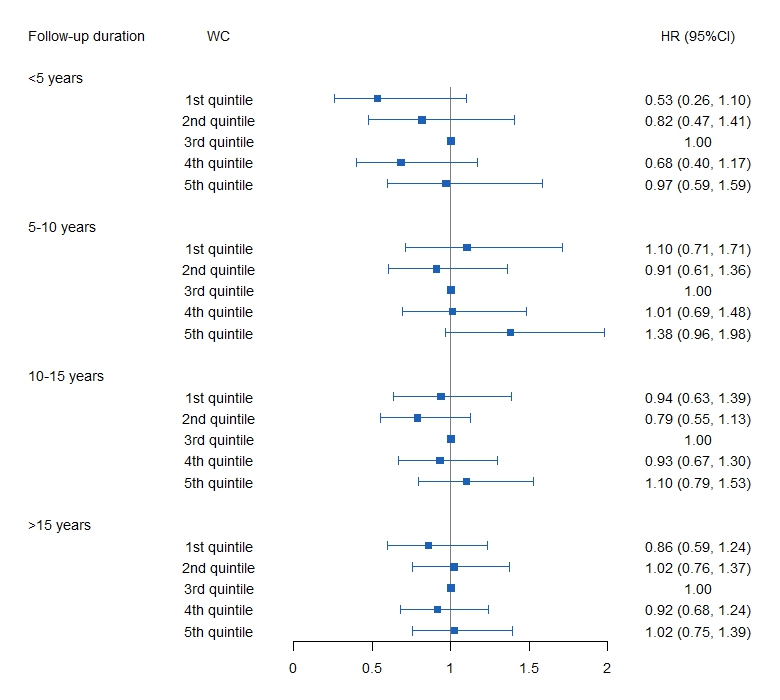


Figure S6. Hazard ratios (HR) for mortality in association with WHR stratified by follow-up duration. Models were adjusted for age, sex, marital status, education, smoking, alcohol drinking, leisure time physical activity, anti-hyperglycemic drugs, fasting glucose, hypertension, uric acid, cardiovascular disease (including stroke), chronic kidney disease, and cancer. Abbreviations: WHR, waist-hip ratio; CI, confidence interval.

1. All-cause mortality


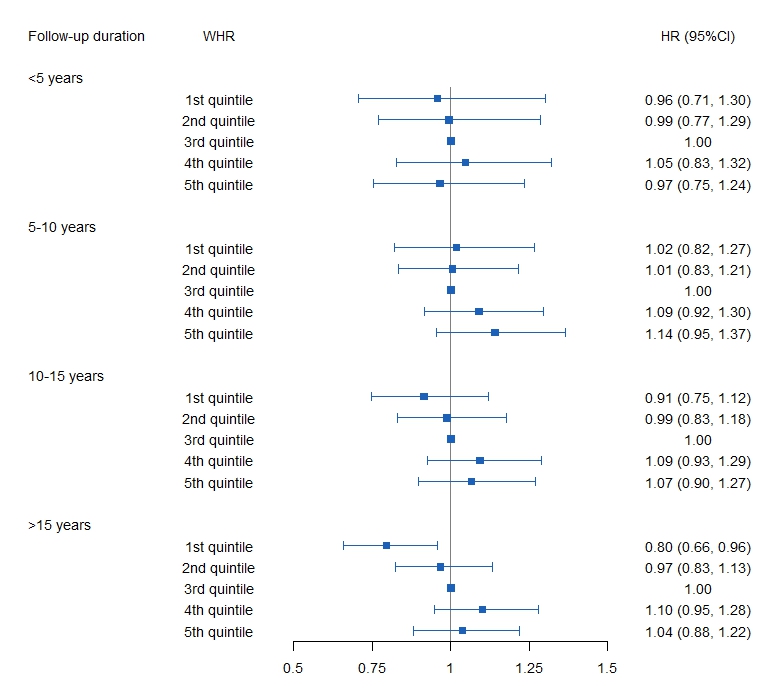


1. Cardiovascular mortality


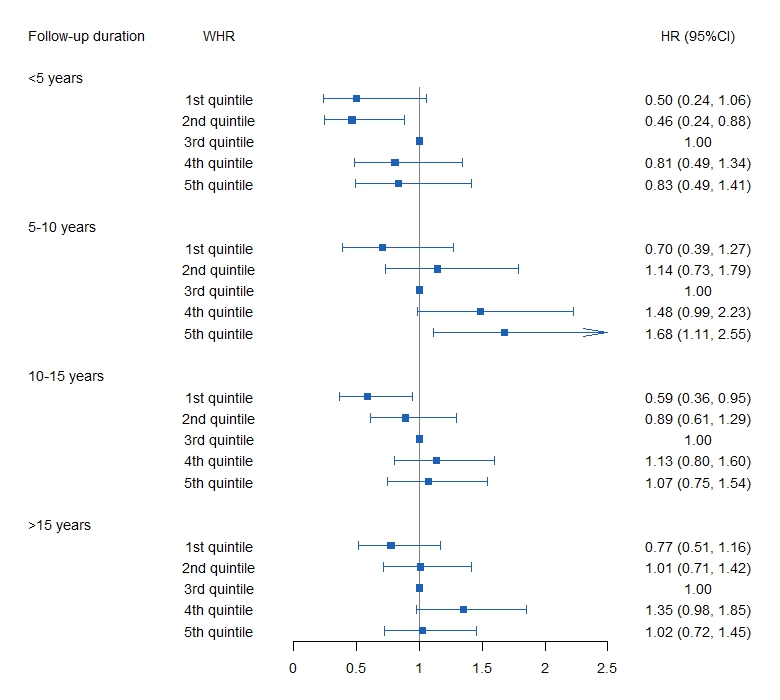


Figure S7. Hazard ratios (HR) for mortality in association with BFP stratified by follow-up duration. Models were adjusted for age, sex, marital status, education, smoking, alcohol drinking, leisure time physical activity, anti-hyperglycemic drugs, fasting glucose, hypertension, uric acid, cardiovascular disease (including stroke), chronic kidney disease, and cancer. Abbreviations: BFP, body fat percentage; CI, confidence interval.

1. All-cause mortality


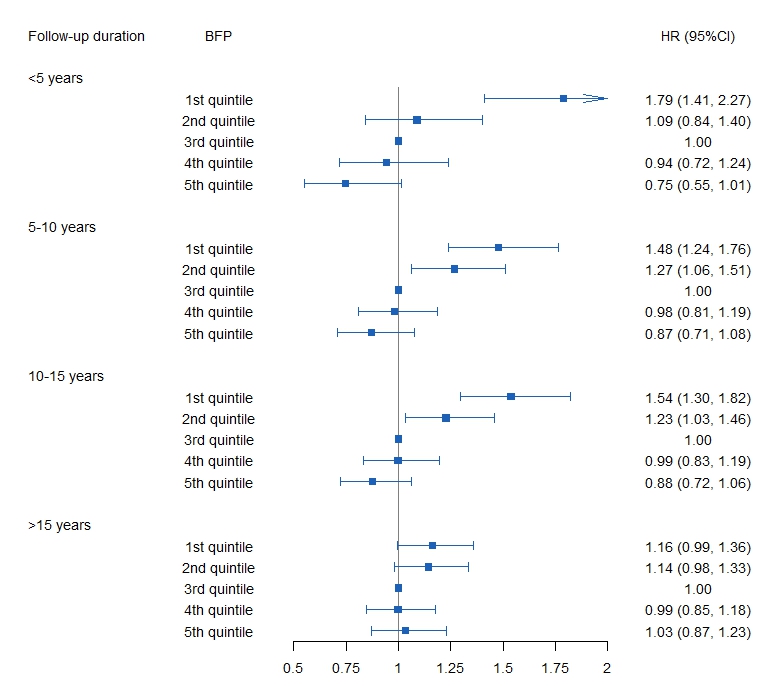


1. Cardiovascular mortality


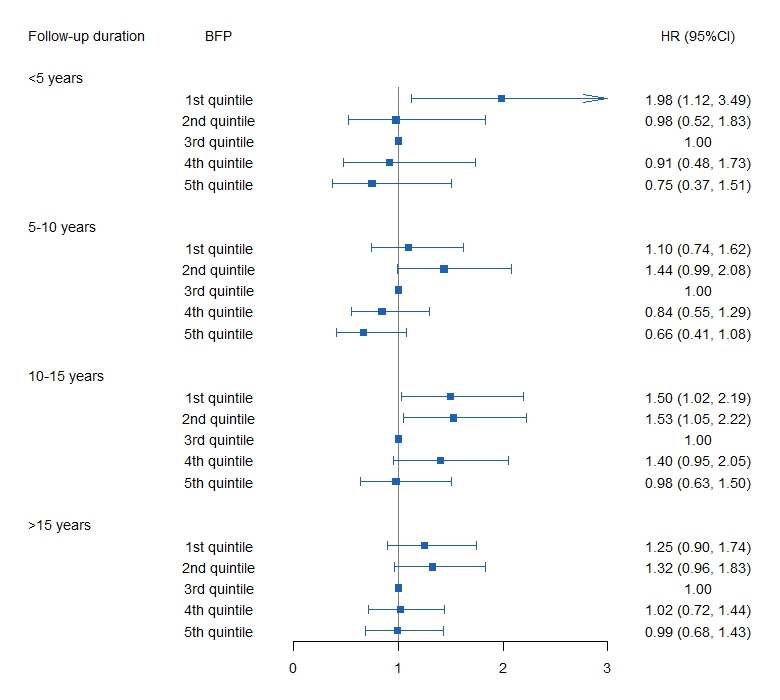


Figure S8. Hazard ratios (HR) for mortality in association with ABSI stratified by follow-up duration. Models were adjusted for age, sex, marital status, education, smoking, alcohol drinking, leisure time physical activity, anti-hyperglycemic drugs, fasting glucose, hypertension, uric acid, cardiovascular disease (including stroke), chronic kidney disease, and cancer. Abbreviations: ABSI, A Body Shape Index; CI, confidence interval1

1. All-cause mortality


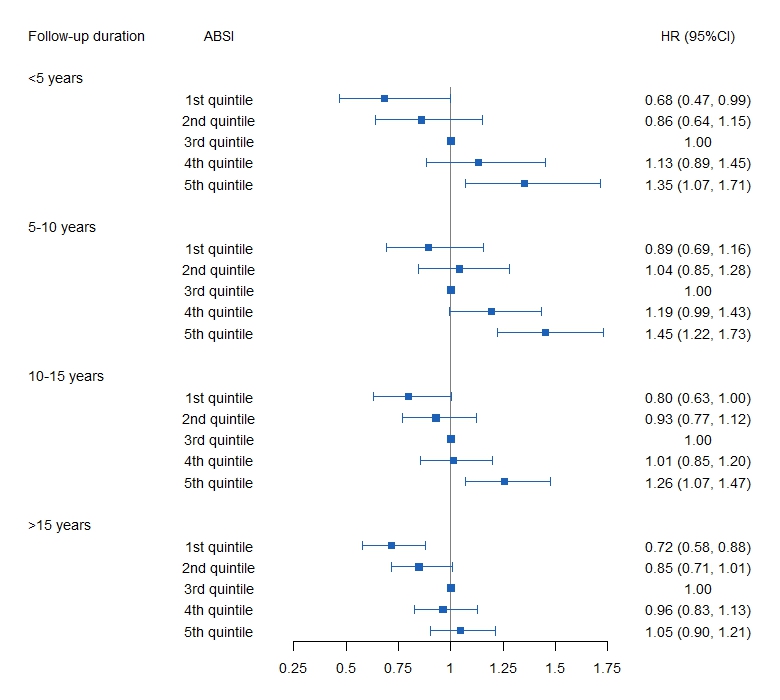


1. Cardiovascular mortality


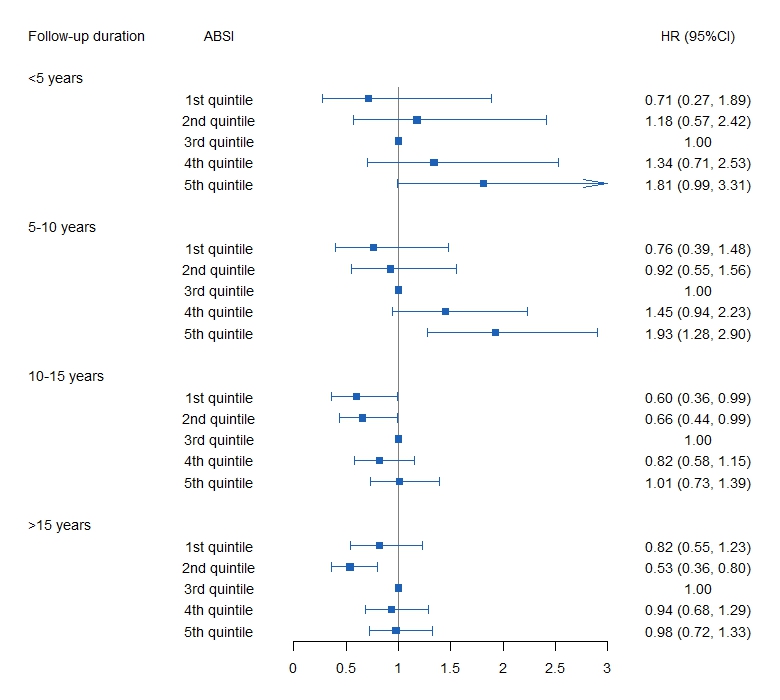

Supplement: Supplementary file 1 — Supplementary Material 1 [file 12933_2023_2072_MOESM1_ESM.docx]
